# Leveraging the Polymer Glass Transition to Access Thermally Switchable Shear Jamming Suspensions

**DOI:** 10.1021/acscentsci.2c01338

**Published:** 2023-03-08

**Authors:** Chuqiao Chen, Michael van der Naald, Abhinendra Singh, Neil D. Dolinski, Grayson L. Jackson, Heinrich M. Jaeger, Stuart J. Rowan, Juan J. de Pablo

**Affiliations:** † Pritzker School of Molecular Engineering, University of Chicago, Chicago, Illinois 60637, USA; ‡ Department of Physics, The University of Chicago, Chicago, Illinois 60637, USA; § James Franck Institute, The University of Chicago, Chicago, Illinois 60637, USA; ∥ Department of Macromolecular Science and Engineering, Case Western Reserve University, Cleveland, Ohio 44106, USA; ⊥ Department of Chemistry, The University of Chicago, Chicago, Illinois 60637, USA; # Center for Molecular Engineering, Argonne National Laboratory, Lemont, Illinois 60439, USA

## Abstract

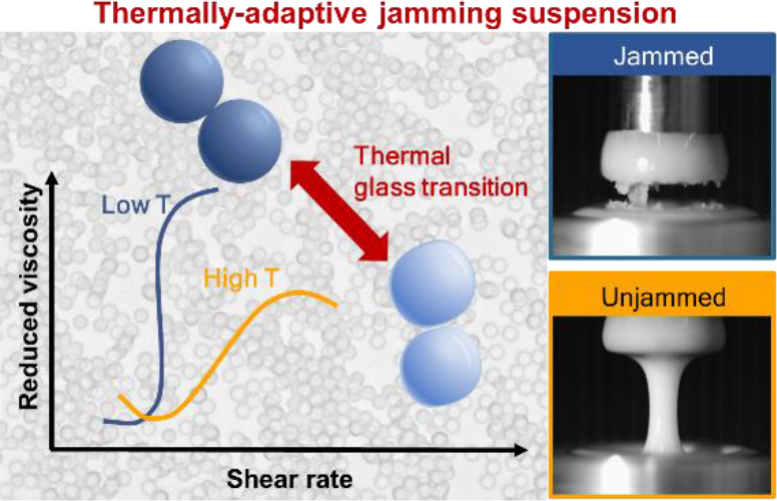

Suspensions of polymeric nano- and microparticles are fascinating stress-responsive material systems that, depending on their composition, can display a diverse range of flow properties under shear, such as drastic thinning, thickening, and even jamming (reversible solidification driven by shear). However, investigations to date have almost exclusively focused on nonresponsive particles, which do not allow *in situ* tuning of the flow properties. Polymeric materials possess rich phase transitions that can be directly tuned by their chemical structures, which has enabled researchers to engineer versatile adaptive materials that can respond to targeted external stimuli. Reported herein are suspensions of (readily prepared) micrometer-sized polymeric particles with accessible glass transition temperatures (*T*_g_) designed to thermally control their non-Newtonian rheology. The underlying mechanical stiffness and interparticle friction between particles change dramatically near *T*_g_. Capitalizing on these properties, it is shown that, in contrast to conventional systems, a dramatic and nonmonotonic change in shear thickening occurs as the suspensions transition through the particles’ *T*_g_. This straightforward strategy enables the *in situ* turning on (or off) of the system’s ability to shear jam by varying the temperature relative to *T*_g_ and lays the groundwork for other types of stimuli-responsive jamming systems through polymer chemistry.

## Abstract 2

Suspensions consisting of polymer particles that can undergo glass transition are designed to enable the *in situ* turning on (or off) of the system’s ability to shear jam by varying the temperature.

## Introduction

The structure–property relationships established for polymers have enabled the design of adaptive materials that can undergo rapid changes upon exposure to an external stimulus such as temperature, pH, or light.^1−4^ Such adaptive material systems have been developed for a wide range of applications spanning both macroscopic and microscopic scales. In particular, micro- and nanoparticles made from responsive polymers have drawn attention from diverse fields,^4^ including drug delivery,^5^ optical systems,^6^ and rheological control.^7^ In these systems, careful selection of polymer chemistries enables the precise control of single-particle properties at the microscopic level. This highly tunable approach makes it possible to regulate the collective behaviors in colloids comprising such particles, such as their macroscopic packing and various transport properties.

Dense suspensions consist of a large volume fraction (ϕ) of small solid particles dispersed in a carrier fluid. Such materials display rich rheological behaviors and provide intriguing opportunities to design material systems with applications complementary to solid polymeric materials.^8−13^ On account of the prevalence of complex interactions that involve interparticle contact forces in addition to hydrodynamic coupling forces, dense suspensions can display drastic non-Newtonian rheology that in extreme cases resembles a reversible liquid to solid transition.^11,14−20^ As the contact interactions are highly sensitive to the composition and surface structures of the particles, the stress response of the suspension can be tailored by varying the fluid and the particle mechanical properties.^7,21−26^ For example, suspensions consisting of soft particles (*E*′ = 10^2^–10^4^ Pa) can exhibit dramatic shear thinning and provide platforms for designing novel lubricants.^10−13^ On the other hand, dense suspensions of rigid particles (typically glassy materials with Young’s moduli *E*′ = 10^8^–10^11^ Pa) can exhibit a dramatic increase in viscosity or even solidify under shear (shear jamming), thereby finding applications in impact mitigation.^8,9,16,18,27−29^ While suspensions consisting of diverse classes of materials have been investigated to map out the structure–property relationships that connect particle properties to suspension rheology, material systems that allow for *in situ* control of suspension flow behavior have received limited attention.^7,22,30,31^ One prototypical example is provided by microgel suspensions of soft thermoresponsive particles.^7,25,30^ In such systems, the lightly cross-linked polymer network can be driven to deswell through triggering a solubility transition at certain temperatures, leading to a drastic decrease in the volume fraction of the particles and a decrease in the suspension yield stress under shear.^23^ While this strategy is effective for tuning shear thinning, systems relying on solubility transitions are limited to a few selected solvents.

In contrast to solubility transitions, the polymer glass transition can result in a dramatic change in particle stiffness without the aid of a solvent. As one of the most fundamental properties of polymers, a considerable amount of research has been aimed at characterizing polymeric interfaces around the glass transition temperature (*T*_g_). When a polymeric material is heated through *T*_g_, within a span of just a few degrees the molecular mobility increases by over 10 orders of magnitude,^32−34^ elastic moduli drop by 2–3 orders of magnitude,^35−37^ and friction exhibits a distinct temperature anomaly.^38−44^ Remarkably, while these changes around *T*_g_ potentially have a large influence on the interparticle forces in dense suspensions, their role in controlling, and tailoring, the suspension flow behavior remains largely unexplored due to a lack of experimental studies in the vicinity of *T*_g_.

This work considers the interplay between polymer glass formation/devitrification and shear-jamming phenomena and how that can be leveraged to arrive at suspensions with adaptable shear-jamming characteristics. More specifically, introduced herein is a generally applicable strategy that relies on the design of suspensions of particles with targeted *T*_g_ to access materials with responsive and switchable shear rheology. It is demonstrated that transitioning through *T*_g_ has a dramatic and nonmonotonic effect on the shear thickening strength of the suspension. This behavior enables the *in situ* turning on (or off) of the material’s ability to shear jam by varying the temperature relative to *T*_g_ and lays the groundwork for switchable jamming systems that leverage a diverse range of polymer chemistry.

## Results and Discussion

A vast majority of polymeric particles reported in the dense suspension literature are based on poly(styrene) (PS) or poly(methyl methacrylate) (PMMA), which exhibit a glass transition temperature (*T*_g_) that is well above ambient conditions (*T*_g_ ∼ 100 °C). Such temperatures are rarely accessed in rheological measurements.^45,46^ Furthermore, non-cross-linked PS or PMMA particles can suffer from irreversible plastic deformations and dissolution above *T*_g_,^47,48^ and randomly cross-linked polymers can exhibit broadened *T*_g_’s that can be difficult to characterize.^49−51^ These and other features serve to underscore that studying the effect of *T*_g_ in suspension rheology requires the careful choice of the particles’ chemistry.

**Figure 1:**
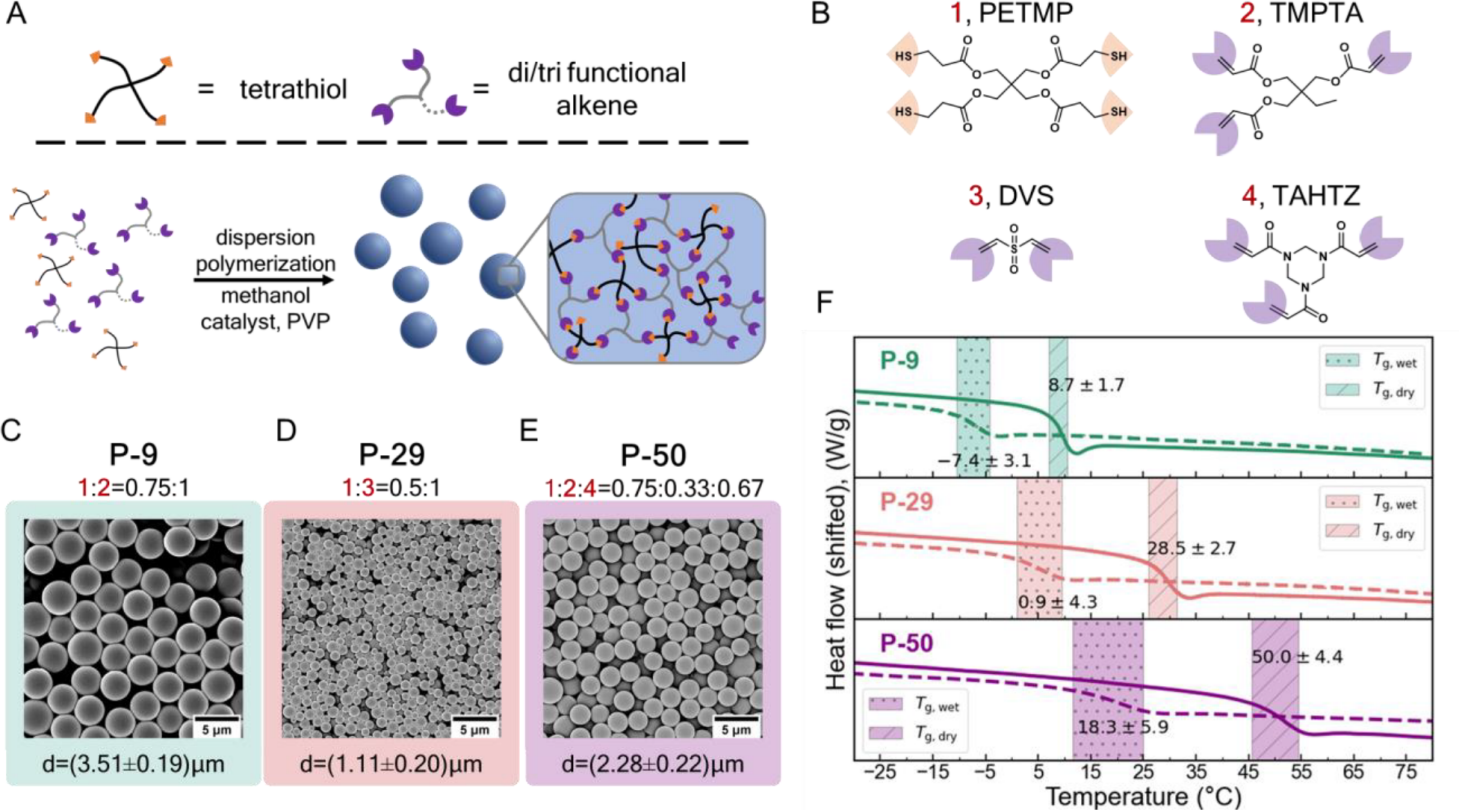
Particle synthesis scheme and characterization by SEM and DSC. (A) Schematic diagram of the thiol-Michael dispersion reaction and the particle network structure. Detailed reactions and purification conditions are in Materials and Methods in the Supporting Information. (B) Chemical structures of the monomers, namely, pentaerythritol tetrakis(3-mercaptopropionate) (PETMP, **1**), trimethylolpropane triacrylate (TMPTA, **2**), divinyl sulfone (DVS, **3**), and 1,3,5-triacryloylhexahydro-1,3,5-triazine (TAHTZ, **4**), used to synthesize the different particles. (C–E) Monomer composition and SEM micrographs of **P-9**, **P-29**, and **P-50** particles, respectively. The mean size and the standard deviation are indicated below each image. (F) DSC measurement results showing the normalized heat flow as a function of *T* for the dry particles (solid line) and particles suspended in their respective carrier fluid at around 56 wt % (dashed lines). Shaded areas with (·) and (/) indicate the wet and dry *T*_g_, respectively. The first number is the half-height midpoint of the glass transition (*T*_g_) range. The second value indicates the width of the *T*_g_ range.

To access a series of cross-linked particles with a range of distinct and accessible *T*_g_’s, polymeric particles were synthesized using thiol-Michael dispersion polymerization^52−54^ of small-molecule monomers (Figure 1A and B). This technique offers several advantages: (1) the product is a polymer network with a high cross-linking density and minimal plastic deformation above *T*_g_, (2) the glass transition temperature range is narrow, (3) the *T*_g_ of the polymer can be precisely tuned by varying the monomer structures, and (4) the reaction is facile and relatively insensitive to moisture or air.^52−54^ Three types of particles (referred to as **P-*X***, where *X* is the dry *T*_g_ in °C), namely, **P-9**, **P-29**, and **P-50** were synthesized by thiol-Michael dispersion polymerization at a stoichiometric thiol/vinyl ratio following general procedures reported by Bowman and co-workers.^52,53^ In each case, a tetrathiol, namely, pentaerythritol tetrakis(3-mercaptopropionate) (PETMP, **1**), was mixed with monomer(s) containing multiple Michael acceptor functionalities, namely, trimethylolpropane triacrylate (TMPTA, **2**), divinyl sulfone (DVS, **3**), or 1,3,5-triacryloylhexahydro-1,3,5-triazine (TAHTZ, **4**), at a vinyl to thiol ratio of 1:1. By varying the type and ratio of the different Michael acceptor-containing monomers, it was possible to tailor the *T*_g_ of the particles (Figure 1C–E). The reaction was monitored by vinyl/thiol conversion via FTIR spectroscopy (see Figure S1–S3). Images from scanning electron microscopy (SEM) demonstrate that the particles were uniform in size (Figure 1C–E and Figure S4).

To create suspensions, poly(ethylene glycol) of molecular weight (*M*_*n*_) 200 g/mol (PEG200) was used as the carrier fluid for all the particle systems (in the case of the **P-29** particles, 20 vol % dimethyl sulfoxide was added as a cosolvent to assist with dispersion, see the Materials and Methods in the Supporting Information for further details). The *T*_g_’s of both the dry particles and the suspensions were determined by differential scanning calorimetry (DSC), as shown in Figure 1F. In all three cases, the *T*_g_ of the particles immersed in the carrier fluid drops by 15–25 °C below that of the dry particles, suggesting that the carrier fluid acts as a plasticizer that can accelerate the polymer dynamics in the bulk and on the surface.^26^ Nevertheless, the particle dimensions stay roughly the same in the dry or wet state on account of their high cross-linking density (see Figure S5).

**Figure 2:**
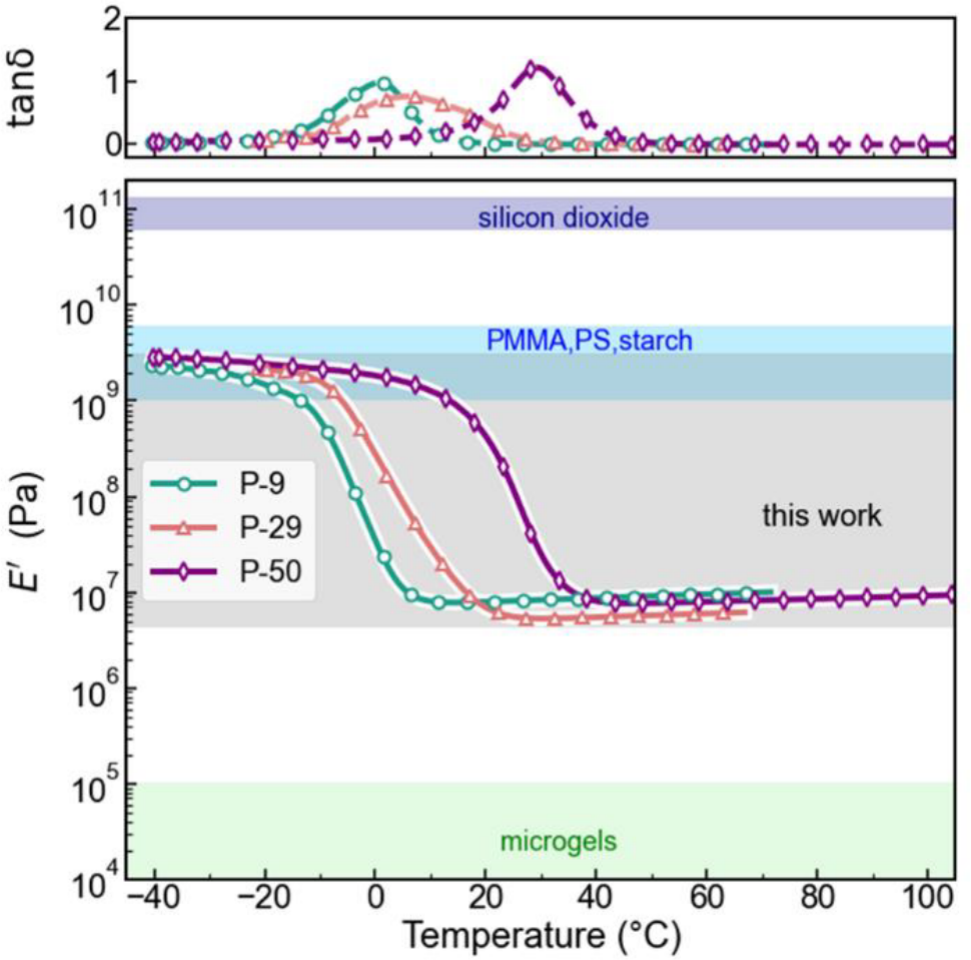
Mechanical stiffness characterization via DMA. The storage modulus (*E*′) and tan δ = *E*″/*E*′ of the carrier-fluid-swollen **P-9**, **P-29**, and **P-50** polymer films were measured in an immersion setup using an oscillation frequency of *f* = 1 Hz. See Figure S6 for plots including the loss modulus (*E*″). The thermomechanical *T*_g_ is indicated by the peak in tan δ. The values of *E*′ for several other particle materials are indicated for comparison.^57−60^ PMMA = poly(methyl methacrylate) and PS = polystyrene.

As the thermomechanical properties of these networks represent a critical aspect of this work, they were evaluated by preparing monolithic **P-9**, **P-29**, and **P-50** films, which were characterized by dynamic mechanical analysis (DMA) in both the dry (see Figures S6 and S7) and carrier-fluid-swollen states (Figure 2) for a better representation of the suspension environment. As expected, the polymer networks synthesized using this chemistry exhibit a relatively narrow glass transition window, consistent with literature examples.^52,53^ The ratio of loss to the storage modulus (tan δ) provides a measure of the mechanical losses due to dissipation at the molecular scale.^44,55^ In general, the peak in tan δ is seen around *T*_g_ because polymer chains just start to become mobile at that temperature but still experience high molecular friction, leading to higher dissipation than in the glassy or rubbery states. The thermomechanical *T*_g_ of the materials, designated by the peak in the dissipation factor tan δ, is comparable to that from the DSC measurements for the dry and immersed film (see Figures S6 and S7). Importantly, the tensile storage modulus (*E*′) changes by three orders of magnitude from around 10^9^ Pa in the glassy state to 10^6^ Pa in the rubbery state for all three materials.

To examine the effect of temperature (*T*) on the rheology, steady-state flow curves for particle suspensions with fixed particle volume fractions were measured at different temperatures as a function of shear stress (τ). The suspension viscosity η can be normalized by the viscosity η_0_(*T*) of the Newtonian carrier fluid to define a relative viscosity η_r_ = η/η_0_(*T*) (see Figure S8). Critically, the strength of shear thickening can be parametrized by the power law exponent β (η_r_ ∝ τ^β^), which represents the slope of the curve during the shear thickening regime on a log–log plot, with β = 0 corresponding to a Newtonian fluid and β = 1.0 signaling the occurrence of discontinuous shear thickening (DST), which is a precursor of shear jamming in steady-state rheological measurements.^8,16,19,56^

**Figure 3:**
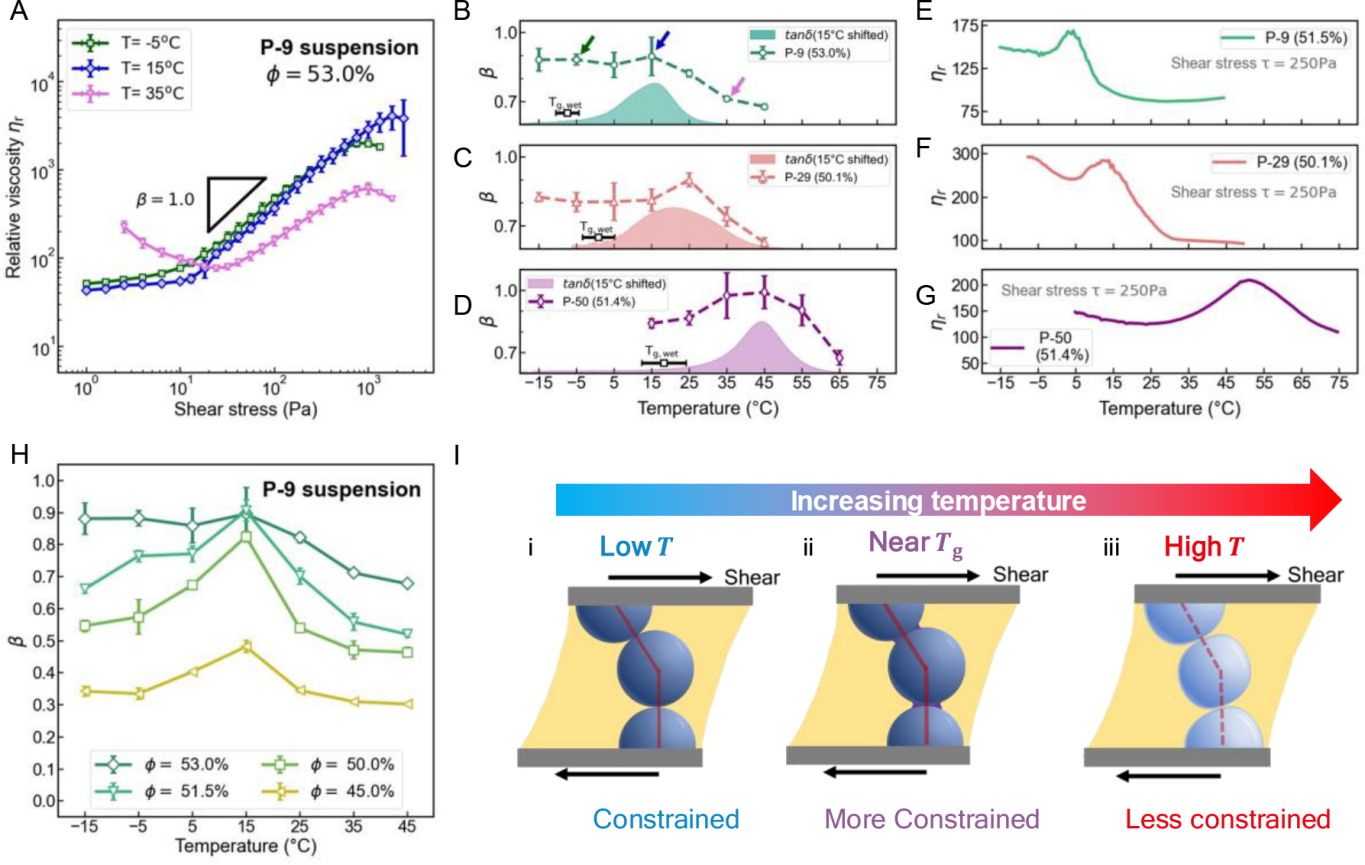
Dependence of the rheological behavior on temperature. (A) Stress-controlled steady-state rheometry data for the **P-9** suspension with ϕ = 53.0 vol % at −5 (green), 15 (blue), and 35 °C (pink), demonstrating that the strength of shear thickening is highly temperature-dependent. The black line has a slope of 1 corresponding to DST where the shear rate is constant. (B–D) β as a function of the 53.0% **P-9** suspension, the 50.1% **P-29** suspension, and the 51.4% **P-50** suspension. Shaded areas indicate the tan δ results from Figure 2, here shifted up by 15 °C. The unshifted *T*_g_ values from the DSC measurement of the suspension are indicated by the black line. Arrows in panel B indicate β for the conditions measured in panel A. (E–G) Reduced viscosity η_r_ measured as a function of temperature for the 51.5% **P-9** suspension, the 50.1% **P-29** suspension, and the 51.4% **P-50** suspension at a shear stress of 250 Pa. (H) Plots of the shear thickening exponent β (η_*r*_ ∝ τ^β^) as a function of temperature for four packing fractions of **P-9** suspensions. Error bars represent the standard deviation from three or more replicate measurements in panels A–D and H. (I) Illustration of the proposed temperature dependency mechanism. (i) Below *T*_g_, the particles are glassy and nondeformable. (ii) The particles exhibit maximum constraints near *T*_g_ due to greater frictional interactions between the polymer particles. (iii) At temperatures high above *T*_g_, surface deformability dominates and the particles are less constrained. The deformations are small, as estimated from contact mechanics calculations (see Figure S11), and they are exaggerated in this illustration for visual clarity. Flow curves and plots of η_*r*_ vs the shear rate can be found in Figure S9–S12.

In typical suspensions of hard spheres, such as silica particles, the η_r_ vs τ curves are independent of temperature, and for a given packing fraction β is roughly constant (with an estimated decline of less than 0.03 per 10 °C).^19,61^ In contrast to the behavior of these traditional suspensions, the **P-9** particle suspension shows a strong sensitivity to temperature, as demonstrated in Figure 3A, where η_r_ for a volume fraction of ϕ = 53% shows a significant change in shear thickening strength as *T* is varied from −15 to 45 °C. Here, a strong and nearly discontinuous thickening with β ≈ 0.9 was observed below 15 °C. These findings should be contrasted with measurements at higher temperatures, for example 35 and 45 °C, where the suspension shows only mild thickening (β < 0.7) (Figure 3B and Figure S9). Interestingly, note that the secondary thinning that follows the thickening, as predicted by Jamali and Wagner, was not observed, which could be a consequence of limitations on the shear rates accessible by parallel plate rheometers.^62,63^ The change in β over a temperature range of ∼20 °C suggests that control of shear thickening should be possible simply by modulating temperature, thereby providing new avenues for engineering responsive fluids or for facilitating suspension processing, which is typically challenging without the use of additives in the carrier fluid.

As an alternative to measuring β vs *T*, a common way to probe molecular dynamics in a polymer and molecular glass transition is to investigate how viscosity varies with temperature for a given stress.^37^ A temperature ramp experiment under constant shear stress (τ = 250 Pa, heating/cooling rate = 1.5 °C/min) was conducted for **P-9**, **P-29**, and **P-50** suspensions (Figure 3E–G and Figure S12). In all cases, the particle volume fraction was held at ϕ ∼ 50%. To normalize the temperature dependency of the carrier fluid viscosity (η_0_), η_r_ = η/η_0_(*T*) is shown in Figure 3E–G. The relative viscosity η_r_ can be regarded as a measure of the additional resistance due to the presence of the particles. For conventional hard sphere suspensions, the curve is expected to be approximately flat with a slight decreasing trend with temperature.^19,61^ For all systems measured in this study, it was found that the relative viscosity of the suspension clearly shows a local peak near each particle’s *T*_g_, which further supports that the maximal shear thickening occurs around the glass transition. Interestingly, the observed behavior in viscosity bears similarities to studies of polymer dispersions that undergo microphase separation, such as that in block copolymer solutions^64^ and polymers around their lower critical solubility temperature.^65^ In those studies, a peak in η is observed at a temperature where the conformation of polymer chain is changing. Here, the peak in η_r_ suggests maximal constraints on the suspension flow. It is worth noting that this peak can be observed in both heating and cooling experiments (Figure S12), implying reversibility in the interactions induced by *T*_g_.

To confirm whether this phenomenon is indeed related to the particles’ *T*_g_, further experiments were carried out on suspensions of **P-29** and **P-50**. While the three types of particles have different sizes (ranging from ca. 1 to 3.5 μm), the effect of particle size is well understood and has been shown to mainly affect the onset shear stress of thickening but not the trend in β.^20,66,67^ For these higher *T*_g_ suspensions, the nonmonotonic behavior in β is found to be similar for all particle compositions. The peak, however, is shifted to higher temperatures, consistent with the changes in *T*_g_ (Figure 3B–D, see Figure S9 for the flow curves). Critically, in all three cases the peak in β can be well aligned with tan δ in Figure 2 when the latter is shifted by the same amount Δ*T* = 15 °C, suggesting that the temperature dependence is directly related to the thermal glass transition of the particles (see Figure S9e and f for a comparison between *T*_g_ and the β peak). An offset Δ*T* may arise from two effects: (1) Polymer glass transition is a kinetic event where the dynamics can start to slow down at temperatures as much as 50 °C higher than *T*_g_(35) and the characteristic dissipation length scale has a measurable increase at more than 15 °C higher than *T*_g_.^42^ (2) Polymer interfaces under confinement and, in particular, with different deformation rates can exhibit dynamics different from the bulk.^34,35^ This result motivates further research to quantify how polymer stress relaxation at the interface can influence the constraints on particle relative motions.^67^ For the three materials considered here, the fact that the maximum observed β strongly tracks with *T*_g_ offers a general yet simple approach to controlling the shear thickening characteristics of dense suspensions by simply tuning the *T*_g_ of the cross-linked particles.

To explore how β vs *T* varies for different volume fractions (ϕ), detailed studies were carried out using **P-9** suspensions at four additional ϕ values that spanned weak to strong shear thickening regimes (see Figure 3H and Figure S10). In all cases, β is a nonmonotonic function of *T*, with a peak near 15 °C for all volume fractions studied (Figure 3H). Above 15 °C, β drops significantly as the temperature increases. At 45 °C, β is smaller than 0.7 even at the highest ϕ studied, implying that DST has been suppressed. At this temperature, the elastic modulus is around three orders of magnitude smaller than in the glassy state. In this rubbery regime, the deformation caused by typical interparticle stress in shear experiments is estimated to be around 1–3% of the particle radius (see Figure S11 for experimental evidence of deformability and additional calculation details). Although this number appears small in magnitude, it is about 100 times larger compared to the deformation in the glassy state and may affect the constraints on particle motions. With this in mind, it is hypothesized that deformability may lead to the observed attenuation in shear thickening at temperatures much above *T*_g_: as the temperature increases, the particle surfaces become more compliant, discouraging the formation of sample-spanning rigid force chains under shear.^62,68^

The observed nonmonotonicity leads us to consider other effects of temperature on the suspension. First, the thermal expansion coefficients (α) of the particle materials were evaluated, since if ϕ increases significantly near *T*_g_ then β should also increase.^19,56^ However, it was found that α of the particles is comparable to that of the carrier fluid (see Figure S13). For all suspensions, the calculated drift in ϕ is minimal (Δϕ < 0.05% for changing *T* by 10 °C) and therefore unlikely to be the main cause of the peak in β (see Figure S14).

Surface friction, which is of vital importance for constraining particle motion, is also extremely sensitive to temperature in the vicinity of *T*_g_.^38−44^ Different from hard surfaces, friction between polymer surfaces originates from dissipative interactions between polymer chains.^39,40,42,44^ Around *T*_g_, adhesive interactions increase on account of an increase in the true contact area, which may lead to stronger cohesive “sticky” forces and enhanced rolling friction.^7,40,41^ On account of the viscoelastic nature of the interface between two particles sheared into contact, the friction coefficient (μ) can either increase monotonically with *T* at moderate to high surface deformation rates or show a peak if the deformation rate is slow enough to cross over into the time scale of surface relaxation^41−44^ (see the Supporting Information for an estimate of the Deborah number). AFM measurements of surface adhesion and friction on dry P-9 films were carried out at varying temperatures with a silicon probe. Consistent with the literature, it was observed that the apparent stiffness decreases while adhesion and friction coefficients increase when heating through *T*_g_ (see Figure S15 for the results and discussion). Increased adhesive interactions above *T*_g_ are consistent with the observation that the **P-9** suspension shows shear thinning at 35 and 45 °C for the low-stress regime (Figure 3A). In relation to shear thickening, simulations and experiments have suggested that both higher friction coefficients μ or the introduction of rolling friction could lead to more pronounced shear thickening and larger β.^7,69,70^

These results suggest that the nonmonotonic trend in the strength of shear thickening with temperature (Figure 3B–D and H) results either from a nonmonotonic μ or from a competition between increasing μ and decreasing mechanical stiffness. In both scenarios, shear thickening is most pronounced when relative particle movement is most constrained. A proposed temperature dependency mechanism is shown in Figure 3*I*.

**Figure 4:**
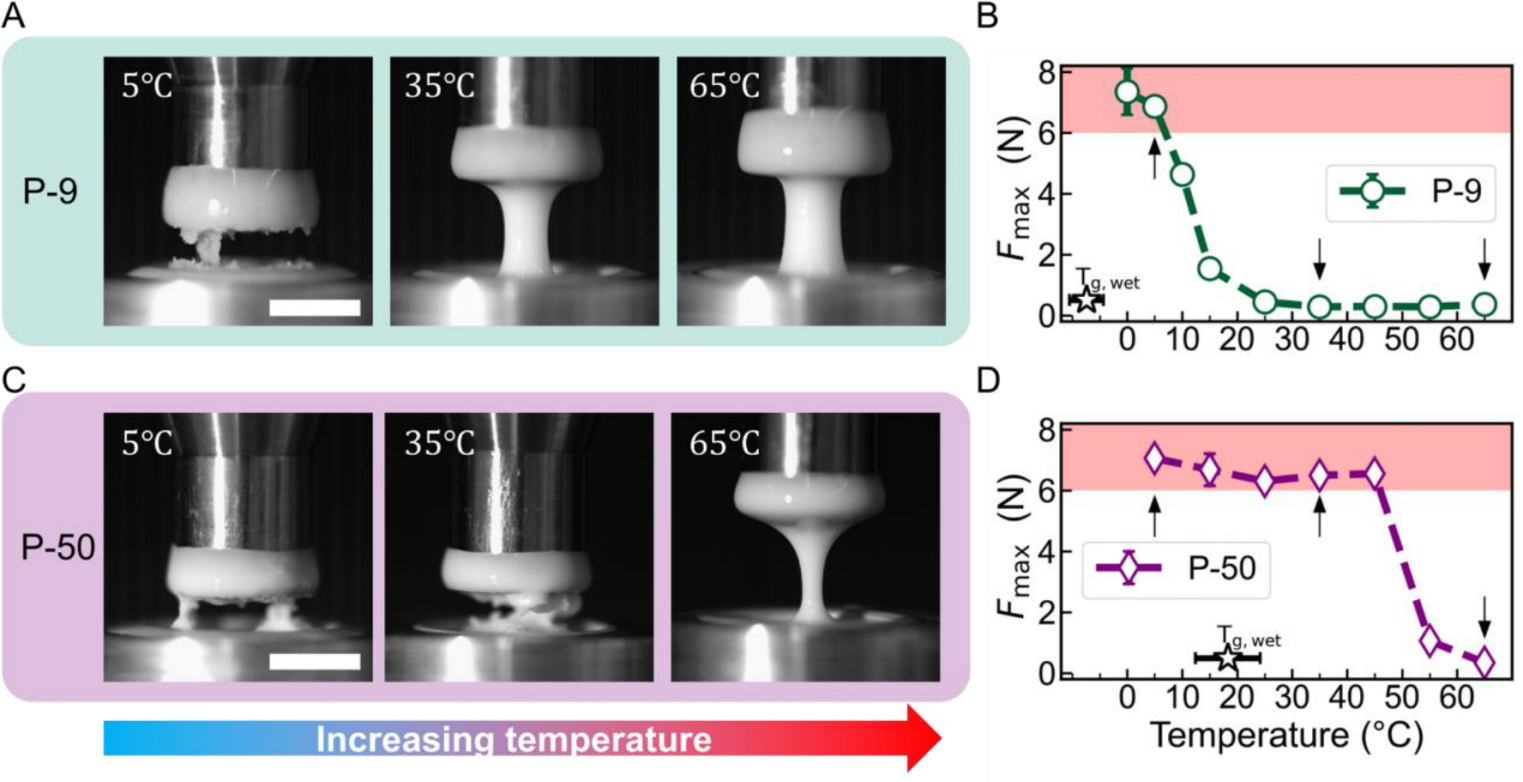
Tensile testing for shear jamming at varying temperatures. (A and C) Images of the suspensions under extensional deformation taken at *T* = 5, 35, and 65 °C for **P-9** and **P-50** suspensions (ϕ = 56.0%). The pulling rate is 8 mm/s. The scale bar indicates 5 mm. (B and D) The maximum normal force (*F*_max_) as a function of temperature. Shaded areas in red indicate large peak normal forces associated with shear jamming. The arrows indicate temperatures corresponding to the snapshots in panels A and C. The unshifted *T*_g_ values from the DSC measurement of the suspension are indicated by the black line. Error bars represent the standard deviation of three replicate measurements. See Figure S15 for representative raw force traces.

Finally, it is important to recall that suspensions that undergo strong or even discontinuous shear thickening in a steady-state rheological measurement may not exhibit shear jamming.^16,24^ To directly assess if our design strategy based on *T*_g_ can be used for switching shear jamming on or off at different temperatures, pull tests^24,71^ were carried out using the **P-9** and **P-50** suspensions. In these experiments, a cylindrical rod, initially immersed in the suspension, is pulled out vertically at a fixed rate, in our case 8 mm/s. At 5 °C, the **P-9** suspension shows a rough cleavage plane, indicating brittle fracture associated with the solid-like behavior of shear-jammed fluids. In contrast, at 35 and 65 °C, the **P-9** suspension exhibits a neck and pinch-off detachment, which is characteristic of a liquid-like response (Figure 4A). The transition between these two types of behavior is also reflected in the maximum normal force *F*_max_ during the deformation, which shows a sharp decrease near 10 °C for the **P-9** system (Figure 4B, see Figure S16 for the raw force curves). In contrast, the **P-50** system shows brittle fracture and large *F*_max_ all the way up to 45 °C, with a liquid-like response occurring only above 45 °C (Figure 4C and D). This clear dependency on *T*_g_ demonstrates how the shear jamming response can be tailored by changing the *T*_g_ of the particles.

## Conclusions

In this study, we have demonstrated that pronounced temperature dependence of the strength of shear thickening in suspensions of polymer microparticles can be achieved by leveraging their glass transition temperature. Most strikingly, the suspensions exhibit a maximal shear thickening near *T*_g_, which is attributed to enhanced frictional or adhesive interactions between the polymer particles sheared into contact. At temperatures above *T*_g_, the particle surfaces become more deformable and do not constrain relative particle movement as strongly and shear jamming can be turned off. Changes in the particle mechanical properties directly impact the force chain formation and, macroscopically, the resistance to flow, as measured by the suspension viscosity (Figure 3I).

In recent years, there has been a growing interest in designing shear thickening fluids (STFs) for targeted material applications.^31,72,73^ Here we provide a different strategy leveraging polymer design based on the bulk mechanical properties of the particle and show how the thermal glass transition in polymers can be used to manipulate STFs. Since *T*_g_ affords wide tunability by altering the chemical structure of the polymer, we believe that this work provides a versatile platform for engineering STFs with tailored mechanical performance. Aside from *T*_g_, there are abundant chemistry designs that have been utilized to synthesize stimuli-responsive colloids.^74−78^ However, very few of these have been investigated from a suspension rheology perspective. Future works that join particle synthesis and suspension rheology can lead to the discovery of new material systems, as well as provide valuable insights into how the microscopic single-particle properties can lead up to the changes in macroscopic flow properties.

From a theoretical perspective, this work raises many exciting questions that warrant deeper investigation. For example, the contact mechanics at a polymer–polymer interface are fundamentally different from those at conventional rigid particle surfaces. It remains to be seen how the interfacial polymer dynamics (i.e., various relaxation times at various length scales) affect the formation and destruction of force chains. Future work that utilizes advanced scattering techniques such as X-ray photon correlation spectroscopy (XPCS) can contribute to a more detailed understanding on the suspension microstructure that bridges single-particle properties with macroscopic rheology and how these microstructures respond to a change in the particle properties.^79,80^

Conversely, given that DST and SJ are exquisitely sensitive to the strength of particle–particle contact interactions, dense suspension rheology provides a powerful lens with which to observe macroscale consequences that are a direct result of interfacial polymer dynamics at molecular length and time scales.

## Supporting Information Available

Experimental methods and materials, suspension preparation, additional rheology data, microscopy images, Deborah number calculation and AFM results (PDF)
**P-9** suspension pull test (AVI)
**P-50** suspension pull test (AVI)

## Supplementary Material

oc2c01338_si_001.pdf

oc2c01338_si_002.avi

oc2c01338_si_003.avi
