# Supplementary material for: Leveraging the Polymer Glass Transition to Access Thermally Switchable Shear Jamming Suspensions
Source: ACS Cent Sci. 2023 Mar 8;9(4):639–47. doi: 10.1021/acscentsci.2c01338 (PMC10141574; doi:10.1021/acscentsci.2c01338)
Supplement: Supplementary file 1 — oc2c01338_si_001.pdf [file oc2c01338_si_001.pdf]

Supplementary materials

Leveraging the polymer glass transition to access thermally-switchable shear jamming suspensions

Chuqiao Chen[1], Michael van de Naald[2], Abhinendra Singh[1,3,4], Neil D. Dolinski[1], Grayson L. Jackson[3], Heinrich M. Jaeger[2,3], Stuart J. Rowan[1,5,6,*], Juan J. de Pablo[1,6,*]

[1]Pritzker School of Molecular Engineering, University of Chicago, Chicago, IL 60637, USA
[2]Department of Physics, The University of Chicago, Chicago, IL, USA
[3]James Franck Institute, The University of Chicago, Chicago, IL 60637, USA
[4]Department of Macromolecular Science and Engineering, Case Western Reserve University, Cleveland, Ohio 44106, USA.
[5]Department of Chemistry, The University of Chicago, Chicago, IL 60637, USA
[6]Center for Molecular Engineering, Argonne National Laboratory, Lemont, IL 60439, USA

* Corresponding author email: SJR: stuartrowan@uchicago.edu; JJdP: depablo@uchicago.edu

Materials and methods S2-S4

List of figures

1 **FT-IR spectra of P-9 particles and monomers.** S5
2 **FT-IR spectra of P-29 particles and monomers.** S6
3 **FT-IR spectra of P-50 particles and monomers.** S7
4 **Histogram of particle sizes from SEM images of P-9, P-29 and P-50 particles.** S8
5 **Swelling of the P-9 particles in PEG200 examined by optical microscopy.** . . . S8
6 **DMA data in dry and wet conditions for the P-9, P-29 and P-50 films.** S9
7 **Overlay of DMA data for the P-9, P-29 and P-50 films in dry conditions.** . . . S10
8 **Viscosity of carrier fluid as a function of temperature.** S10
9 **Rheometry data for P-9, P-29 and P-50 suspensions at varying T.** S11
10 **Steady-state rheology for P-9 particle suspension in PEG200 at different volume fractions ϕ.** S13
11 **Evidence of particle deformability.** S14
12 **Temperature ramp experiments of P-9, P-29 and P-50 suspensions.** S15
13 **Thermal expansion measurement in dry and wet conditions for P-9, P-29 and P-50 films.** S16
14 **Estimation of volume fraction drifts with temperature.** S17
15 **Normal and friction force measurements of P-9 film via AFM.** S18
16 **Representative normal force versus time curves via pull tests.** S21

Deborah number calculations S20

References S22

Materials

Pentaerythritol tetrakis(3-mercaptopropionate) (PETMP) (>95.0%, Sigma-Aldrich), trimethylolpropane triacrylate (TMPTA) (>90%, Sigma-Aldrich), 1,3,5-triacryloylhexahydro -1,3,5 -triazine (TAHTZ, Sigma-Aldrich), divinyl sulfone (>96.0%, TCI America), hexylamine (HEA) (>99.0%, Sigma-Aldrich) and triethylamine (TEA) (>99.5%, Sigma-Aldrich), polyvinylpyrrolidone with average molecular weight 40,000 (PVP) (Sigma Aldrich) were used as received for reactions. Polyethylene glycol with average molecular weight 200 (PEG200) (Sigma Aldrich) and dimethyl sulfoxide (DMSO) (>99.9%, Sigma-Aldrich) were used as received for dispersing the particles. Solvents including methanol, acetonitrile were purchased from Sigma-Aldrich and used as received. PPP-LFMR silicon probes were purchased from Asylum Oxford Instruments.

Methods

Synthesis of P-9 particles. P-9 particles were synthesized via dispersion polymerization following literature procedures. Monomers 5.93 g (20 mmol) of TMPTA and 7.33g (15 mmol) of PETMP were dissolved in 270mL methanol. 3.0g of PVP was dissolved in 30mL methanol and transferred to the reaction flask. The mixture of monomers and surfactant was stirred at 400rpm using a mechanical stir. The reaction was started by adding in the base catalyst hexylamine (0.21g, 1.6wt% relative to monomers) which was diluted by 10 times beforehand in methanol. The reaction was carried out for 4 hours at ambient conditions. The base was neutralized by 1M HCl aqueous solution prior to centrifugation. The dispersion was centrifuged at 1750 m/s^2 for 6 minutes and the supernatant was thrown away. To remove the surfactants and catalyst, the particles were washed in methanol at a concentration of around 0.04 g/mL under sonication and vortexing, followed by centrifugation. This washing process was repeated for 3 times. The particles are dried at 0.2 torr for 48 hours and kept in a desiccator before use. The second batch which was used for OM measurement had a smaller particle size distribution averaged around 3.1μm. For synthesizing the P-9 particles of 2.5μm used for the temperature ramp experiments, triethylamine (4.0wt% relative to monomers) was used instead.

Synthesis of P-29 P-29 particles were synthesized via dispersion polymerization with the same procedure as the P-9 particles with catalyst hexylamine (0.17g, 1.6wt% relative to monomers). Monomers were changed to 3.54g (30 mmol) of DVS and 7.33g (15 mmol) of PETMP.

Synthesis of P-50 particles. P-50 particles were synthesized via dispersion polymerization with procedure similar to the P-9 particles. The molar ratio of TMPTA to TAHTZ was kept 1:2. Specifically, 1.98g (6.67 mmol) of TMPTA, 3.32g (13.33 mmol) of TAHTZ and 7.33g (15 mmol) PETMP was used. Due to the low solubility of TAHTZ monomer at room temperature, the solvent was preheated to 40°C and the monomer was sonicated with the solvent for 1 minute. 0.50g of triethylamine was used as catalyst (4.0wt% relative to the monomers). The rest of the procedures were identical to the synthesis of the P-9 particles.

Scanning electron microscopy. The particles were solvent casted with methanol on silicon wafers and dried in air. Before the SEM analysis, the particles were sputter coated with a 8 nm film of Pt/Pd metal to enhance electron conductivity. The images were recorded on a Merlin SEM (Carl Zeiss) at a voltage 1.5 kV using Inlens detectors and each image was averaged between 8-15 images. Particle sizes were analyzed with ImageJ. Optical microscopy. OM images were taken with an optical microscope (Leica DM2700P) using a 50x objective under transmission mode. Particle suspensions were confined in a channel of around 50μm to avoid compression from the cover slip.

Suspension preparation. Dried particles were dispersed in carrier fluid at certain weight percent-

ages. The weight concentration is converted to volume concentration by the densities of the polymer and the fluid. The mixture was stirred extensively and sonicated for 1 hour. For P-9 and P-50 particles, PEG200 was used as the carrier fluid. For P-29 particles, a mixture containing 80vol% PEG200, 20vol% DMSO and 0.5 wt% NaCl was used to disperse the particles and screen electrostatic interactions.

Polymer film synthesis. Monomers were dissolved in acetonitrile at 30 wt% concentration. Triethylamine was added as a catalyst at a concentration of 0.6 wt% relative to the monomers. After mixing, the solution was immediately injected between two glass slides separated by 1mm spacer. The reaction was carried out overnight in solvent atmosphere. To remove the catalyst, the film was washed with methanol and acetone alternately for 4 times. To evaporate the solvent, the film was put in a vacuum oven at 2 torr and heated at 80 °C for 12 hours and then at 120°C for 4 hours. The catalyst concentration for the P-29 film was reduced by half to counteract the higher reactivity of the DVS monomer. The film sample for AFM measurement was cured with one side facing a silicon wafer to ensure that it is smooth.

Density determination. A piece of polymer film was first immersed in water at 25 °C. 40 wt% NaBr solution was added until the film was exactly levitated. The density of the NaBr solution was then determined by a densimeter (DMA 4500 M, Anton Paar) and designated as the density of the polymer.

FT-IR spectra. Shimadzu IRTracer-100 FT-IR with ATR diamond was used to characterize P-9, P-29 and P-50 particles. Reduction of the thiol peak near 2550cm^{-1} and the alkene peak near 800cm^{-1} were used to monitor the reaction.

DSC measurements. DSC was performed with Discovery 2500 Differential Scanning Calorimeter. 5-10 mg of sample were prepared in aluminum hermetic pans from TA. Typical tests consist of a heat-cool-heat cycle (-40°C to 120°C) at a rate of 5 °C/min.

DMA measurements. Tests were conducted on a RSA-G2 dynamic mechanical analyzer (TA Instruments) equipped with Forced Convection Oven (20 °C -500 °C) attached to an Air Chiller System (-120°C to 20 °C). Samples with thickness of around 0.7 mm were prepared following the method described above and measured under tensile mode. The temperature was ramped at 1.5 °C/min and the oscillatory frequency used was 1 Hz. For measuring the plasticized films, the films were soaked in their respective carrier fluid at 20 °C above T_g for 10 days and the measurement was done while immersed in the carrier fluid. For determination of the thermal expansion coefficients, a constant force 0.1 N was maintained using the isoforce mode and the length (L) change with temperature (T) was recorded. The linear thermal expansion coefficients were extracted by linear fitting to $ln(L)$ v.s. T. $\alpha = 1/L \times d(L)/d(T) = d(lnL)/(d(T))$ in the glassy and rubbery regime.

Shear rheometry measurements. Rheological characterization was performed using an ARES-G2 shear rheometer (TA Instruments) equipped with Forced Convection Oven (20°C to 500°C) attached to an Air Chiller System (−120°C to 20°C). All the rheometry data were acquired with 25mm parallel plates. Change of the gap size due to temperature was calibrated beforehand. The suspension was loaded to the rheometer and extra fluid was trimmed at a gap size of 255μm. The measurement gap size was maintained 240μm throughout the measurement. Temperature was equilibrated for 10 minutes before each measurement followed by a preshearing step at a stress around 2 times of the onset shear stress for 2 minutes. At each stress value, the fluid was equilibrated for a minimum of 30 seconds and averaged over 15 seconds. The first point after preshear was at least equilibrated for 105 seconds and averaged over 15 seconds. Stress controlled measurements were done by looping a series of creep tests on the ARES-G2 rheometer. Temperature ramp experiments were carried out at a ramp rate of 1.5°C/min for both heating and cooling.

Adhesion and friction measurements. AFM (Cypher ES Environmental AFM, Asylum Research Oxford Instruments) was used to measure the adhesion and friction forces. All measurements were carried out in an environmental scanner for temperature control and under nitrogen atmosphere to prevent capillary forces between tip and sample. Tests were performed with PPP-LFMR silicon probes on a smooth and dry P-9 polymer surface prepared with procedures described above. The normal spring constant of the cantilever was determined by the thermal-noise method. The measurements were conducted in accordance with literature procedures [1, 2]. Force distance curves were taken with an approach speed of $2\mu m$/s and the maximum normal load (F_L) was set to 50nN. The adhesion force (F_{ad}) was determined by measuring the pull-off force in the retraction curve. The apparent stiffness (k) was obtained from a linear fit to the extension curve. For the friction measurements, line scans over $5\mu m$ was performed at 1 Hz under varying normal loads between 5nN and 50nN. Care was taken so that the same line was not scanned repeatedly. Friction loops were analyzed using a python script. The normal force F_N is calculated as the sum of normal load and adhesion force (i.e. $F_N = F_L + F_{ad}$). Measurements were repeated for more than 10 scans and the details on the analysis could be found in later section of the SI.

Pull tests. MCR302 rheometer (Anton Paar) was used to measure the pull force following literature procedures[3]. A cylindrical metal cup with 2mm depth and 10mm in diameter was fixed to the bottom plate to contain the suspension. An 8 mm (diameter) rod that was partially embedded to a depth of 1mm in the sample surface and withdrawn at a speed of 8mm/s. Simultaneously, the normal force response on the rod was recorded. Videos were taken with a high-speed camera.

Supplementary Figures and Discussions

a

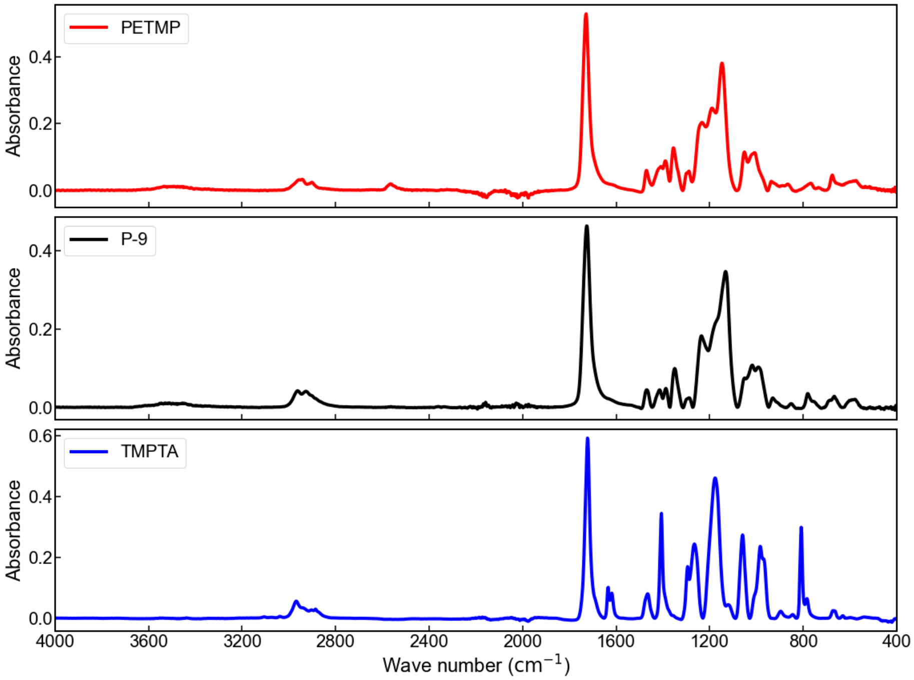

b

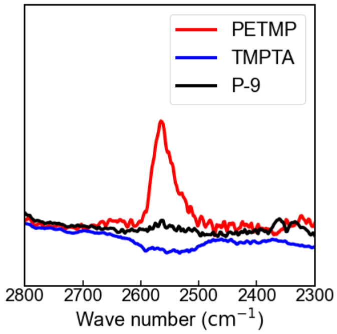

c

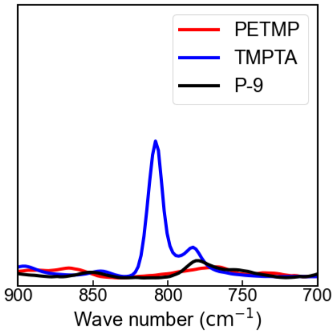

Supplementary Fig. S 1: **FT-IR spectra of P-9 particles and monomers.**
(a) The full spectrum from 4000 cm^{-1}to 400 cm^{-1} is shown. Reduction of the thiol peaks around 2550 cm^{-1} shown in (b) and the C=C peak near 810 cm^{-1} in (c) is consistent with high conversion of the reaction.

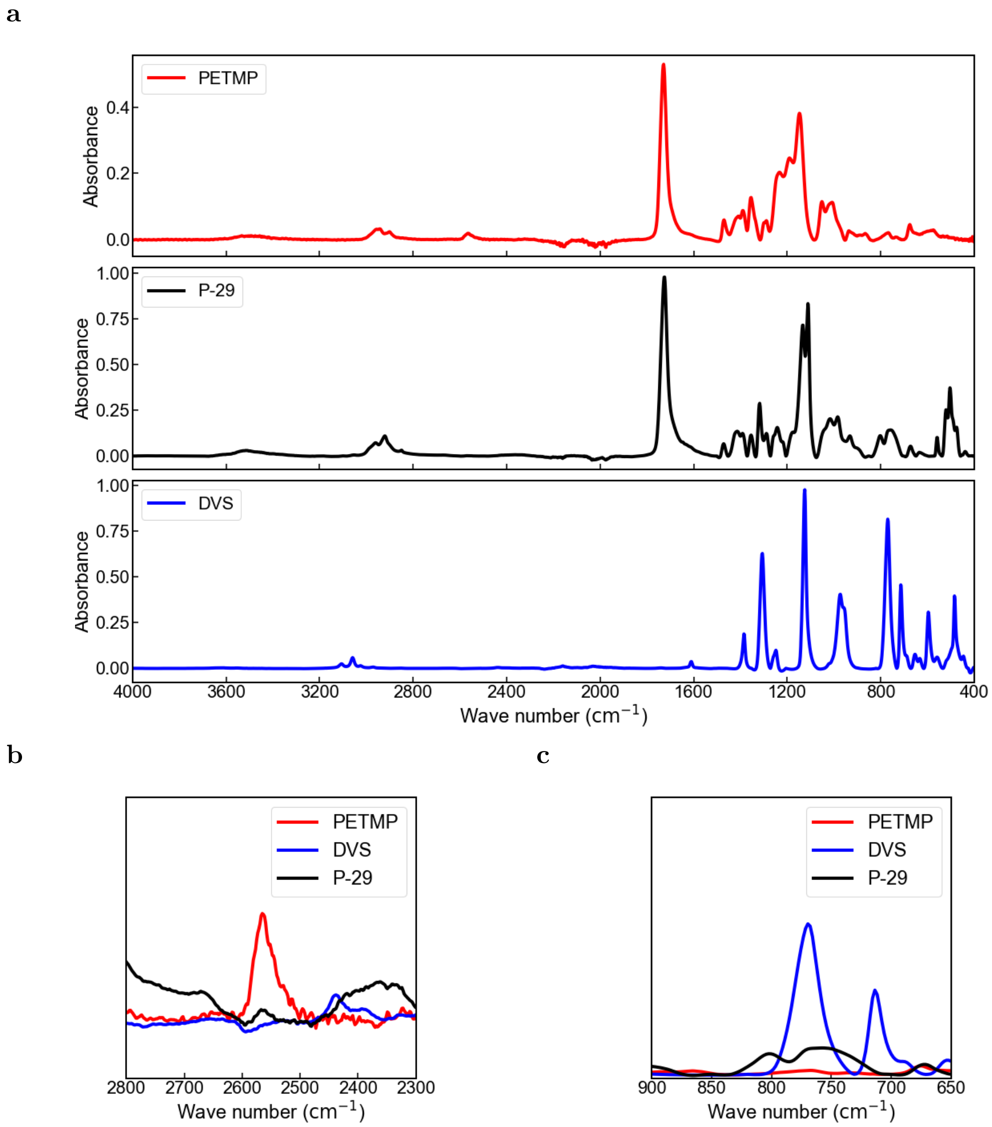

Supplementary Fig. S 2: **FT-IR spectra of P-29 particles and monomers.**
(a) The full spectrum from 4000 cm^{-1}to 400 cm^{-1} is shown. Reduction of the thiol peaks around 2550 cm^{-1} shown in (b) and the C=C peak near 780 cm^{-1} in (c) is consistent with high conversion of the reaction.

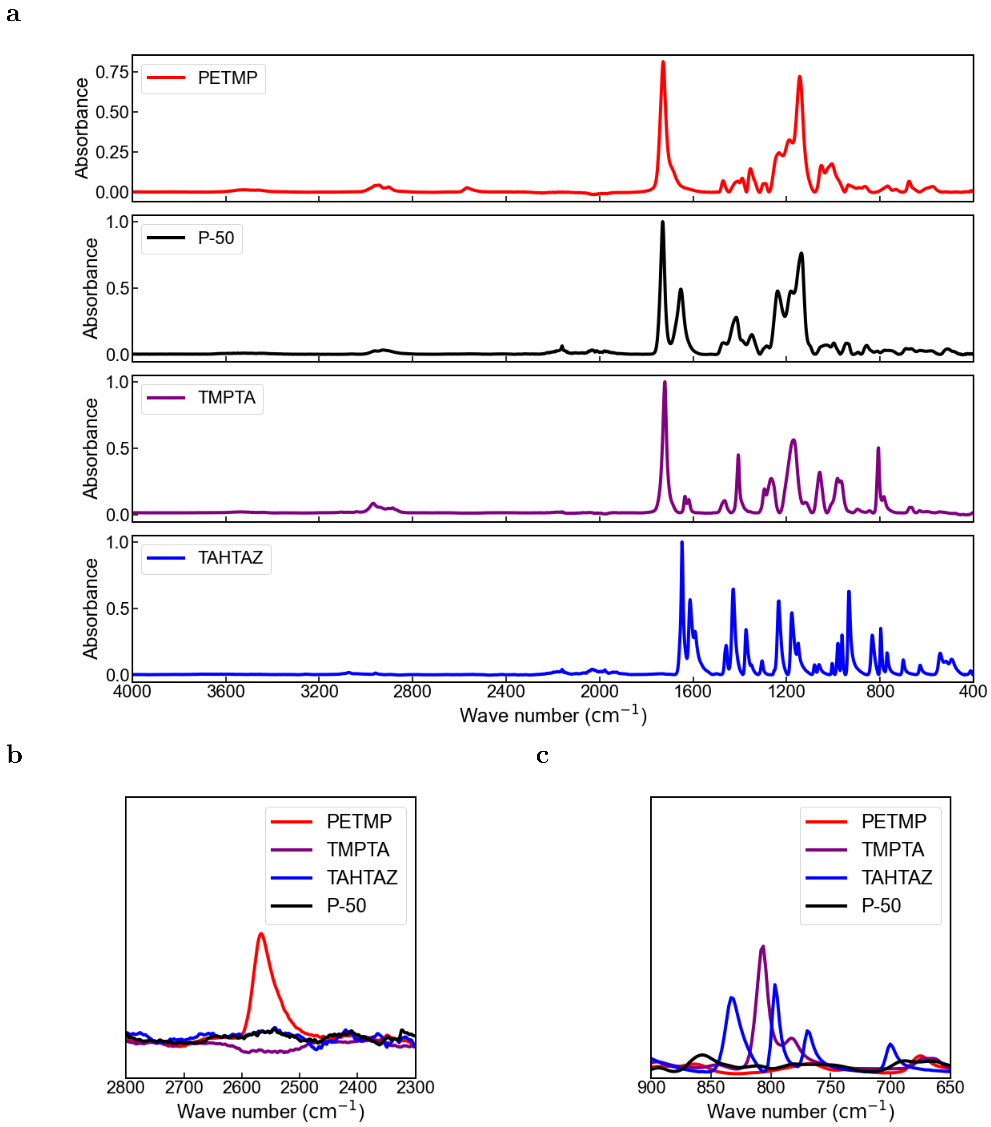

Supplementary Fig. S 3: **FT-IR spectra of P-50 particles and monomers.**
(a) The full spectrum from 4000 cm^{-1}to 400 cm^{-1} is shown. Reduction of the thiol peaks around 2550 cm^{-1} shown in (b) and the C=C peak near 780 cm^{-1} and 810 cm^{-1} in (c) is consistent with high conversion of the reaction.

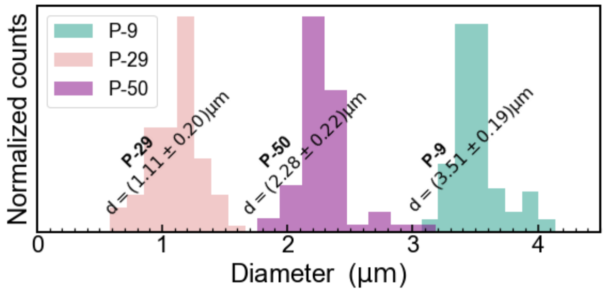

Supplementary Fig. S 4: **Histogram of particle sizes from SEM images of P-9, P-29 and P-50 particles.**

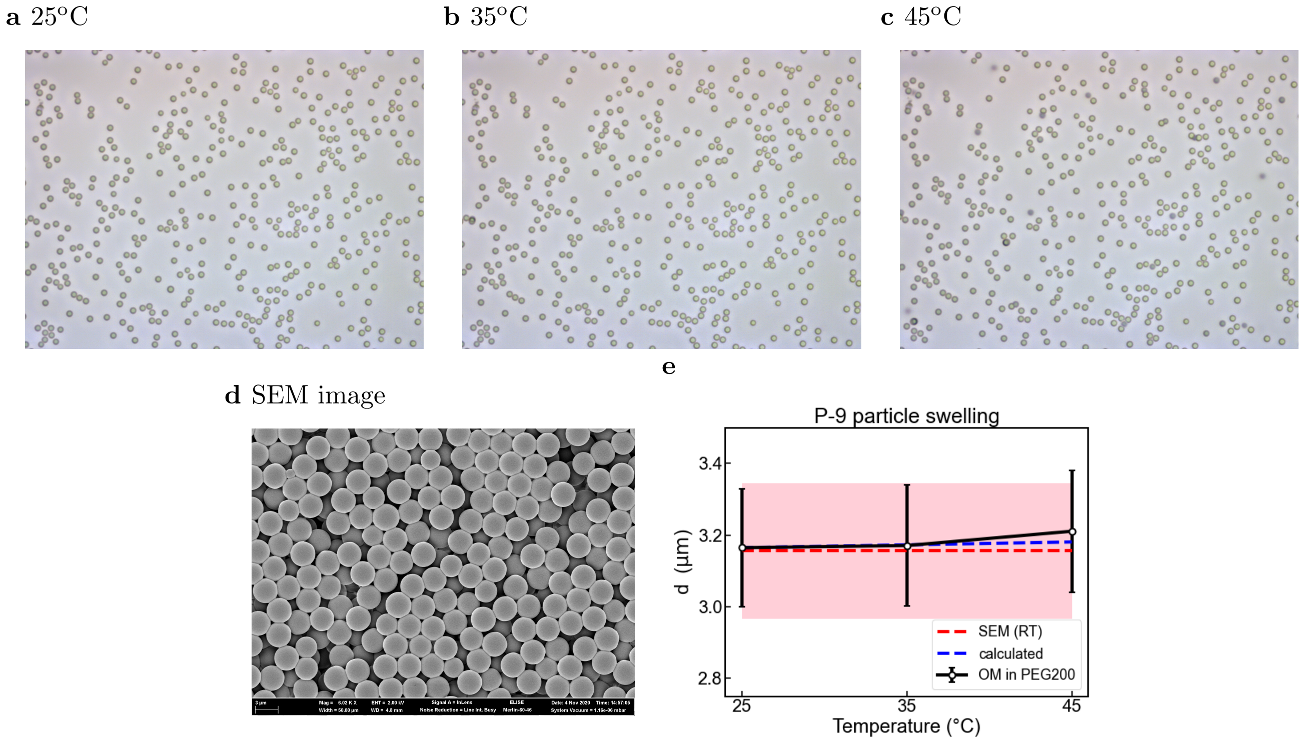

Supplementary Fig. S 5: **Swelling of the P-9 particles in PEG200 examined by optical microscopy.**
(a)-(c) The **P-9** particles were immersed in PEG200 over 24 hours and equilibrated at each temperature for 15 minutes. (d) SEM shows that this batch of **P-9** particles have an average size of $3.16 \pm 0.19\mu$m. (e) The mean size and standard deviation from OM images in (a)-(c) are shown in black line. The error bars represent the standard deviation. Average diameter in (d) is shown in the red dashed line and the pink shaded area represents the standard deviation of the particle size. Calculated diameter using the thermal expansion data of **P-9** is shown by the blue dashed lines.

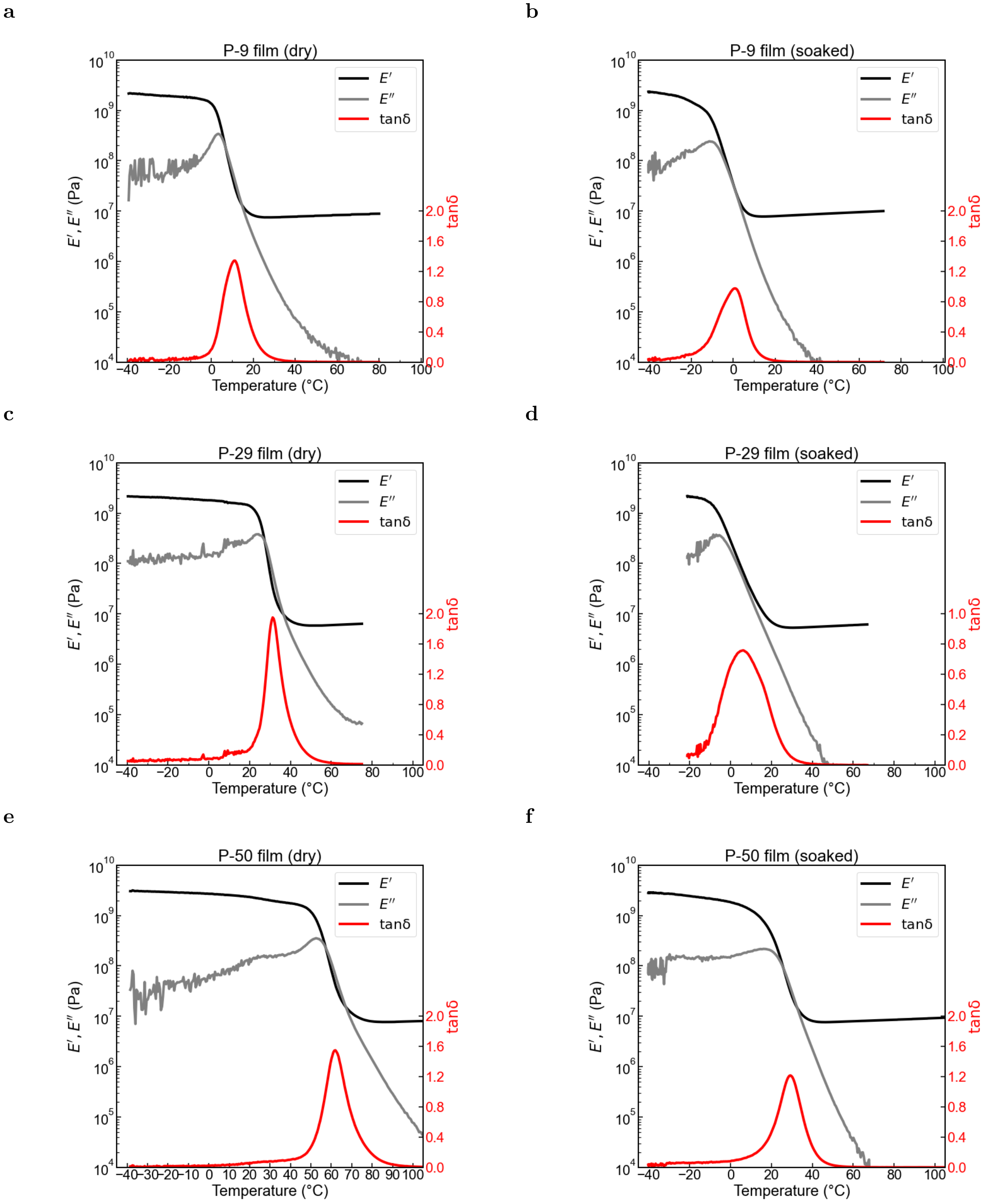

Supplementary Fig. S 6: **DMA data in dry and wet conditions for the P-9, P-29 and P-50 films.**

Oscillation frequency was kept f= 1Hz. The temperature was ramped at 1.5 °C/min. The wet films were soaked in the carrier fluid for 10 days and measured while immersed in the carrier fluid for each of the systems. PEG200 was used for the **P-9** and **P-50** system. A mixed fluid containing PEG200:DMSO=80:20 with 0.5wt% of NaCl was used to for the **P-29** system.

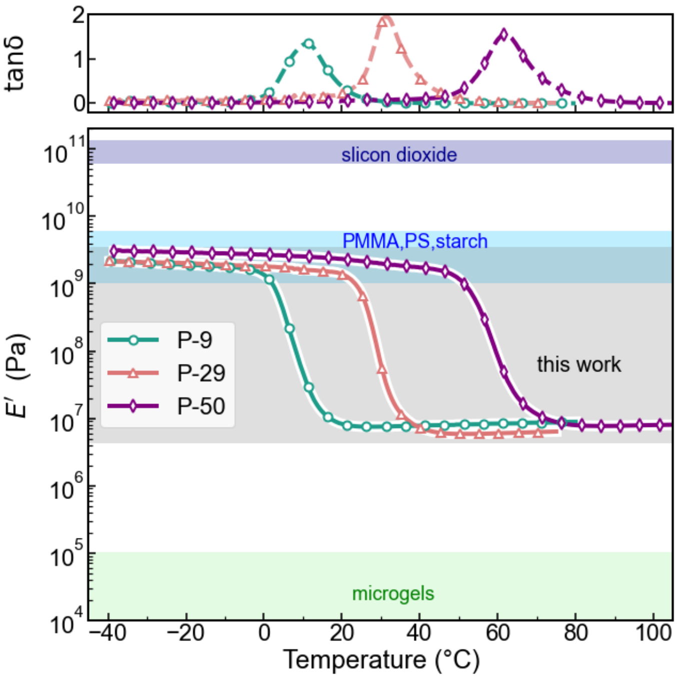

Supplementary Fig. S 7: **Overlay of DMA data for the P-9, P-29 and P-50 films in dry conditions.**
Oscillation frequency was kept f= 1Hz. The temperature was ramped at 1.5 °C/min.

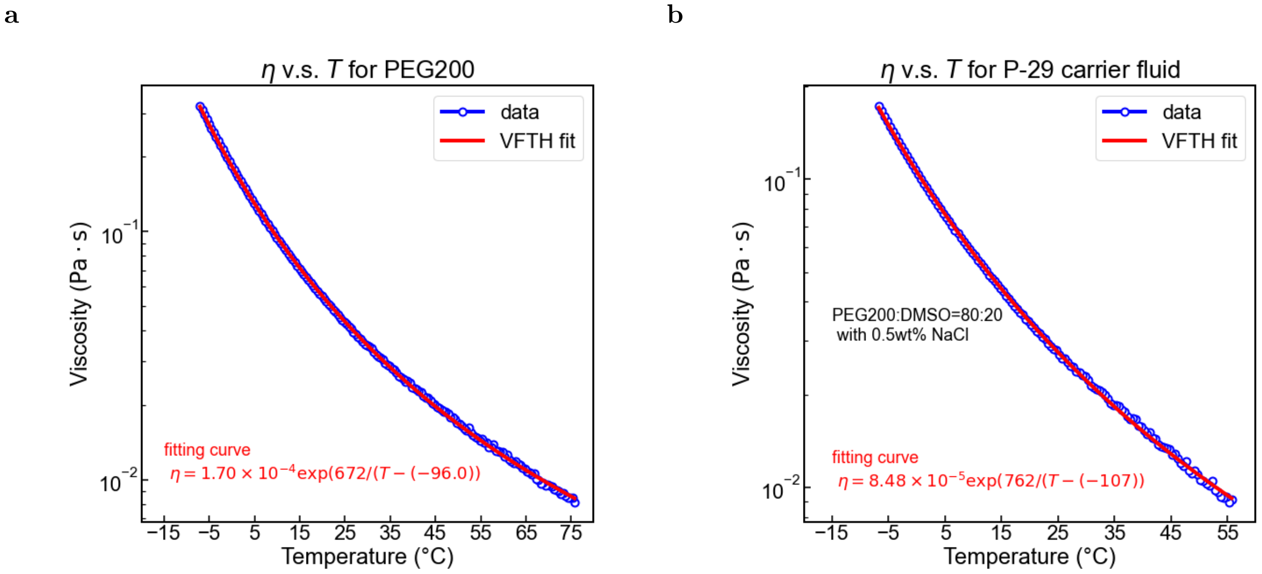

Supplementary Fig. S 8: **Viscosity of carrier fluid as a function of temperature.**
(a) PEG200 was used to suspend the **P-9** and **P-50** particles (b) A mixed fluid containing PEG200:DMSO=80:20 with 0.5wt% of NaCl was used to suspend the **P-29** particles. The temperature ramp experiment was carried at a cooling rate of 1.5°C/min. The viscosity vs temperature was fitted with the Vogel-Fulcher-Tammann-Hesse (VFTH)equation.

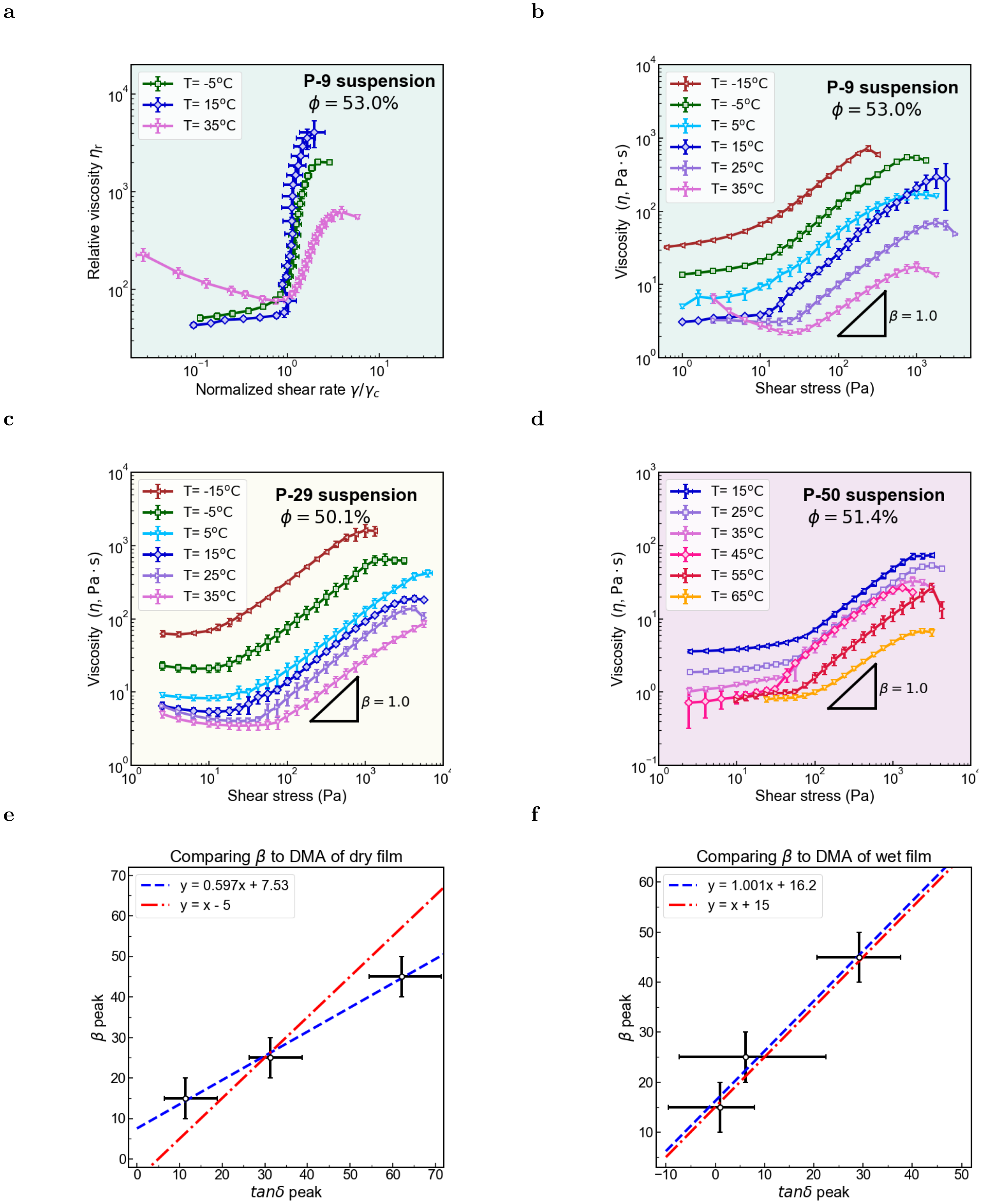

Supplementary Fig. S 9: **Rheometry data for P-9, P-29 and P-50 suspensions at varying T.** (a) η_r vs shear rate for the data shown in Fig. 3a of the main text. The shear rate is normalized by the onset shear rate for better visualization. (b)-(d), The slope β vs T plot is shown in Fig. 3b-d in the main text. Error bars indicate the standard deviation in 3 replicated trials. (e) Correlation between the β beak in (Fig b-d) and dry T_g of the DMA. (f) Correlation between the β beak in (Fig b-d) and wet T_g of the DMA. The x error bars in e and f indicate the width of $tan\delta$ computed from a two-piece Gaussian fit.

Discussion : To determine how the peak in β correlates with the glass transition temperatures, the β peak value is compared to the $tan\delta$ peak value from DMA measurements as shown in Fig. S9 (e-f). In both comparisons, there is a strong positive correlation between the two measurements. Results of linear regression is shown in the blue curve. The optimal solution that constrains the slope to be 1 is shown in the red. It was found that the optimal slope is very close to 1 for Fig. S9(f) which implies that a simple shift of $\Delta T = 15°C$ from the wet T_g can well predict the peak in β for the three systems presented in this work. Note here that the error bar for the x values indicate the width of $tan\delta$ computed from a two-piece Gaussian fit rather than uncertainty.

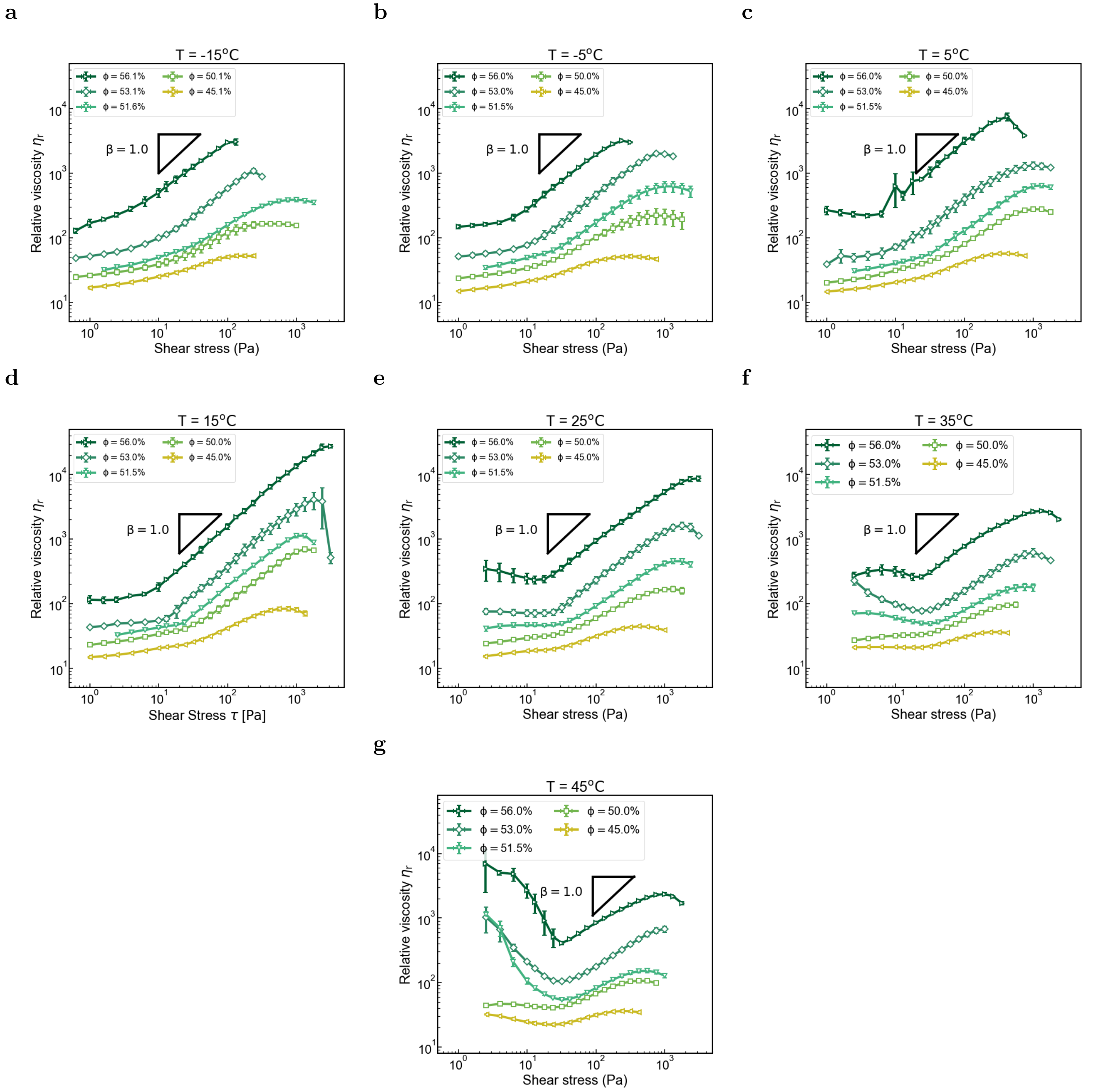

Supplementary Fig. S 10: **Steady-state rheology for P-9 particle suspension in PEG200 at different volume fractions ϕ.**

Measurements are performed at (a) -15°C (b) -5°C (c) 5°C (d) 15°C (e) 25°C (f) 35°C (g) 45°C. Error bars indicate the standard deviation in 3 replicated trials.

a **b**

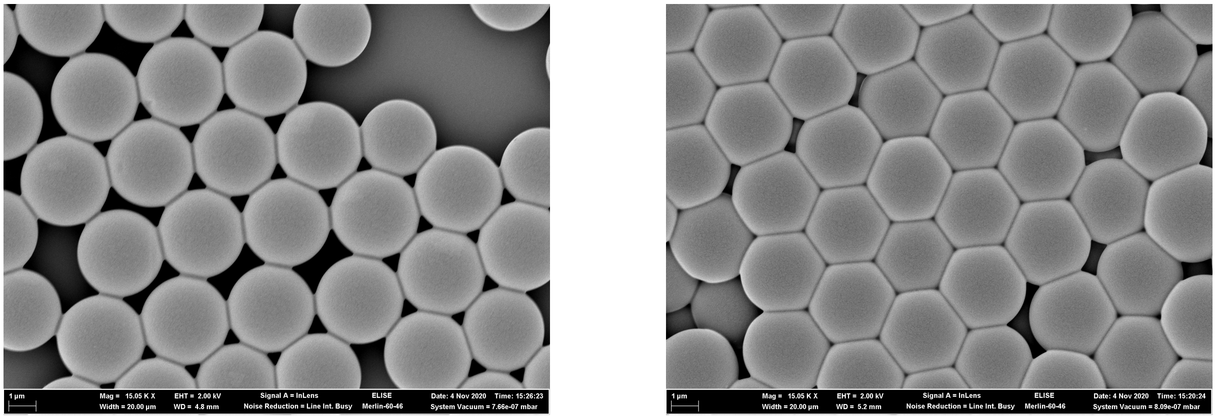

Supplementary Fig. S 11: **Evidence of particle deformability.**
(a) SEM of **P-9** particles in a less packed environment. (b) SEM of **P-9** particles showing deformations due to capillary stress during the drying process.

A discussion regarding the particle deformability above T_g

The above SEM shows that the particles can be deformed by external stress. However, the capillary forces exerted by the solvent casting and drying process is estimated to be 10 to 100 times larger than the contact forces during a shear rheology measurement[4].
If one ignores adhesion forces, the deformation δ for two identical spheres with radius R and elastic modulus E under external force F is estimated by the Hertzian model to be [5]

$$\delta \approx \frac{81F^2}{32E^2R} \tag{1}$$

Above the glass transition temperature, $E \approx 8 \times 10^6$Pa. Assuming that, the largest force exerted on a particle is $10\tau d^2$ where d is the particle radius and τ is the shear stress. (The local shear stress can exceed the boundary stress due to the anisotropy of force chain formations[6, 7]). The numerical values of the maximum deformation is estimated to be:

$$\delta \approx \left(\frac{81\tau^2}{32E^2}\right)^{1/3} R \approx 1 \sim 3\% \tag{2}$$

Although this number appears small, one should keep in mind that the jamming transition is very sensitive to the constraints on particle motions[8]. Another way to view it is in terms of the relative change in E'. The DMA data shows that E' can drop by a factor of $\sim 10^3$ as the materials transition from below to above T_g. From the scaling relation indicated by Eq.2, changing the Young's modulus by 1000 times is effectively changing the deformation by 100 times.

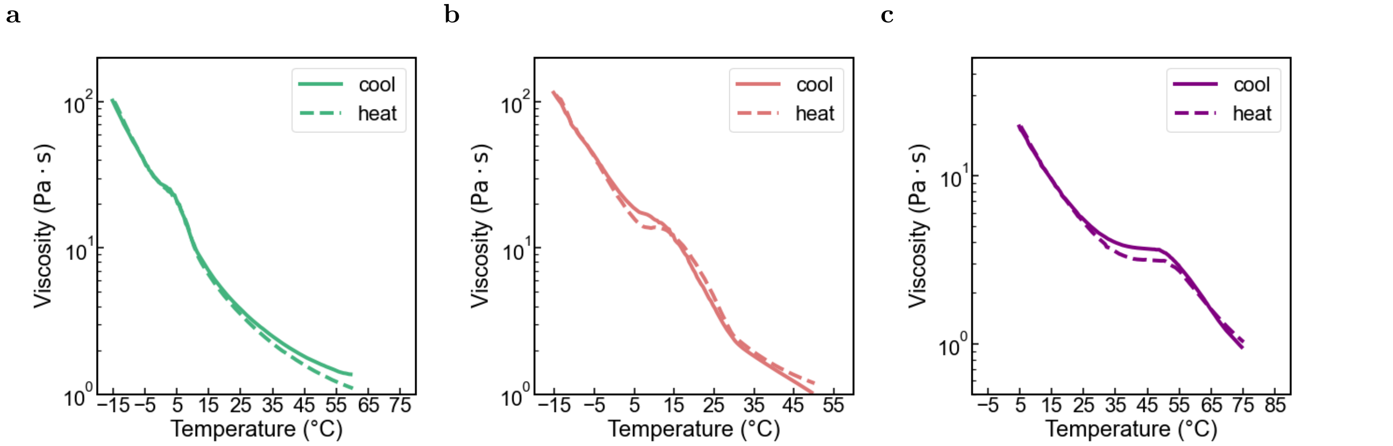

Supplementary Fig. S 12: **Temperature ramp experiments of P-9, P-29 and P-50 suspensions.** In all three cases, the viscosity is measured at constant shear stress of 250Pa. The temperature is first cooled at 1.5°C/min (solid lines) and then heated at 1.5°C/min (dashed lines). The volume fractions are 51.5%, 50.1% and 51.4% for the **P-9**, **P-29** and **P-50** suspensions respectively. The η_r vs T was computed and reported in Fig. 3f-g of the main text.

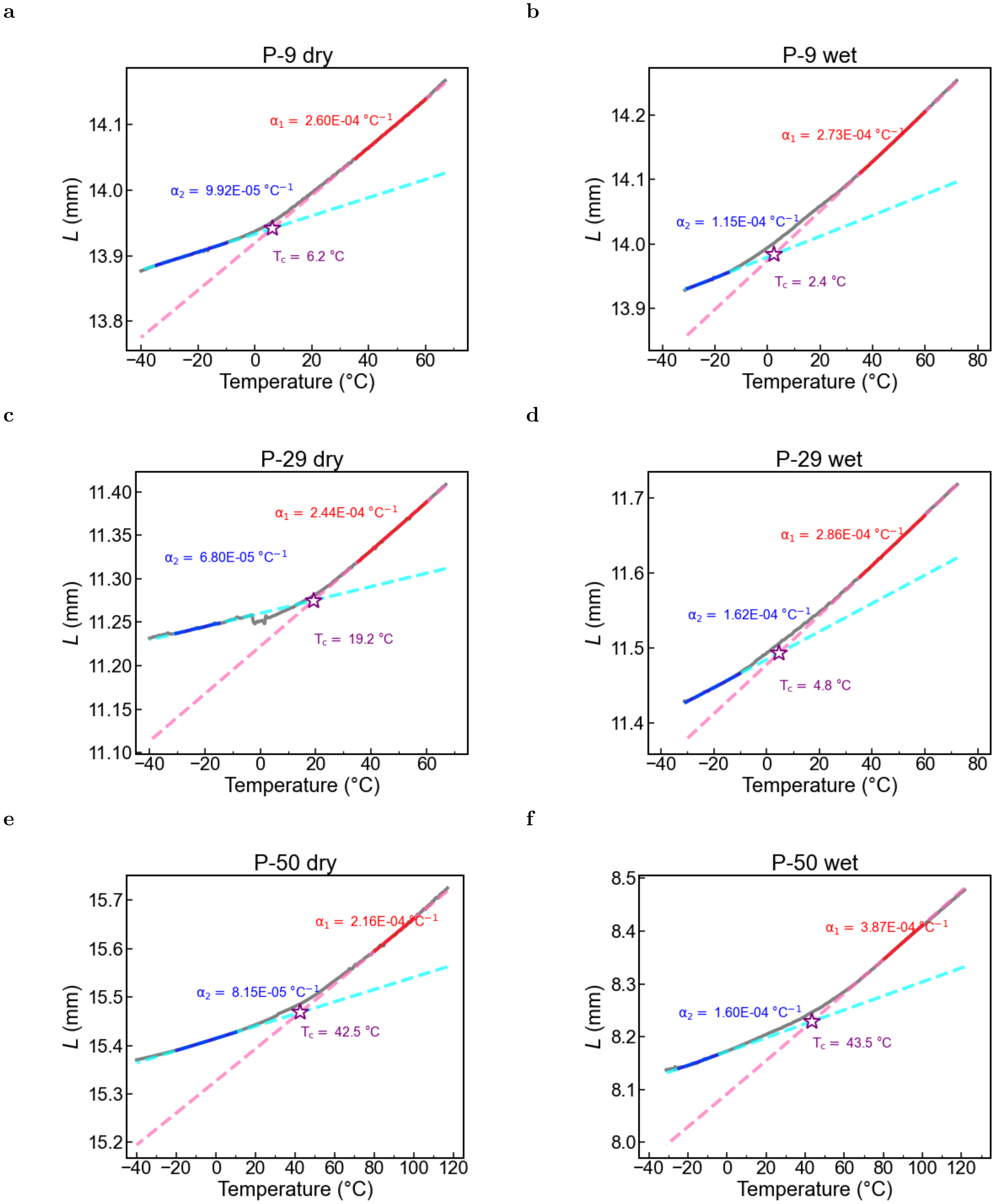

Supplementary Fig. S 13: **Thermal expansion measurement in dry and wet conditions for P-9, P-29 and P-50 films.**

The temperature was ramped at a cooling rate of 1.5 °C/min. The wet films were soaked in the carrier fluid for 10 days and measured while immersed in the carrier fluid for each of the systems. PEG200 was used for the **P-9** and **P-50** system. A mixed fluid containing PEG200:DMSO=80:20 with 0.5wt% of NaCl was used to for the **P-29** system.

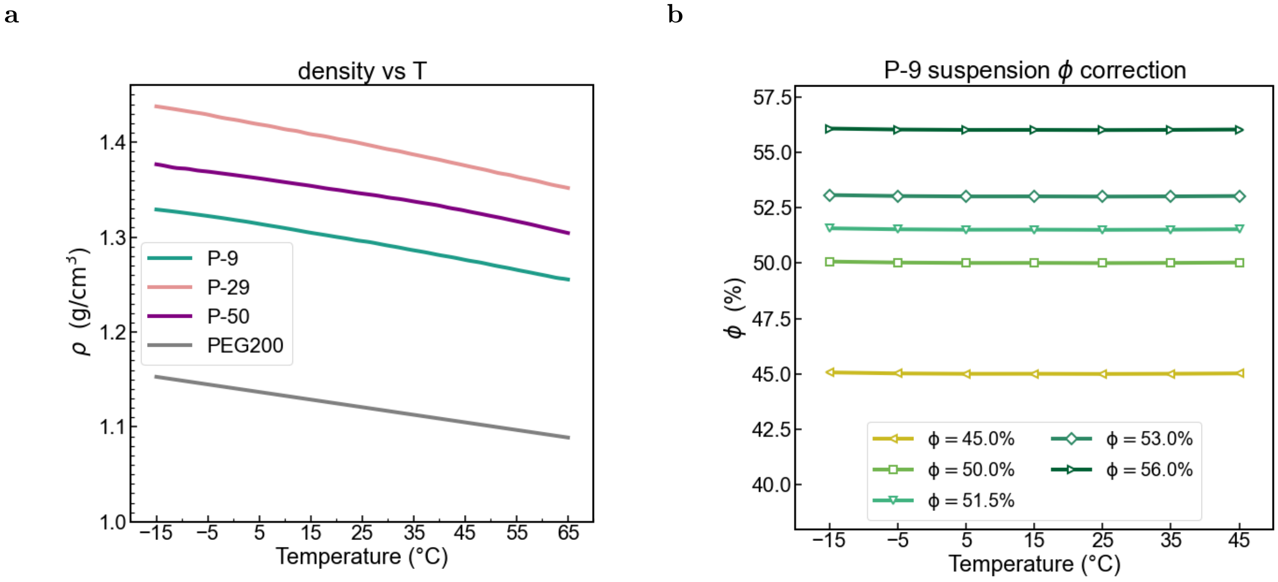

Supplementary Fig. S 14: **Estimation of volume fraction drifts with temperature.**
(a) Estimation of density vs T using the linear thermal expansion coefficient of the soaked films measured in Fig.13. The data for PEG200 is taken from literature[9]. (b)Correction of particle volume fraction in **P-9** suspensions.

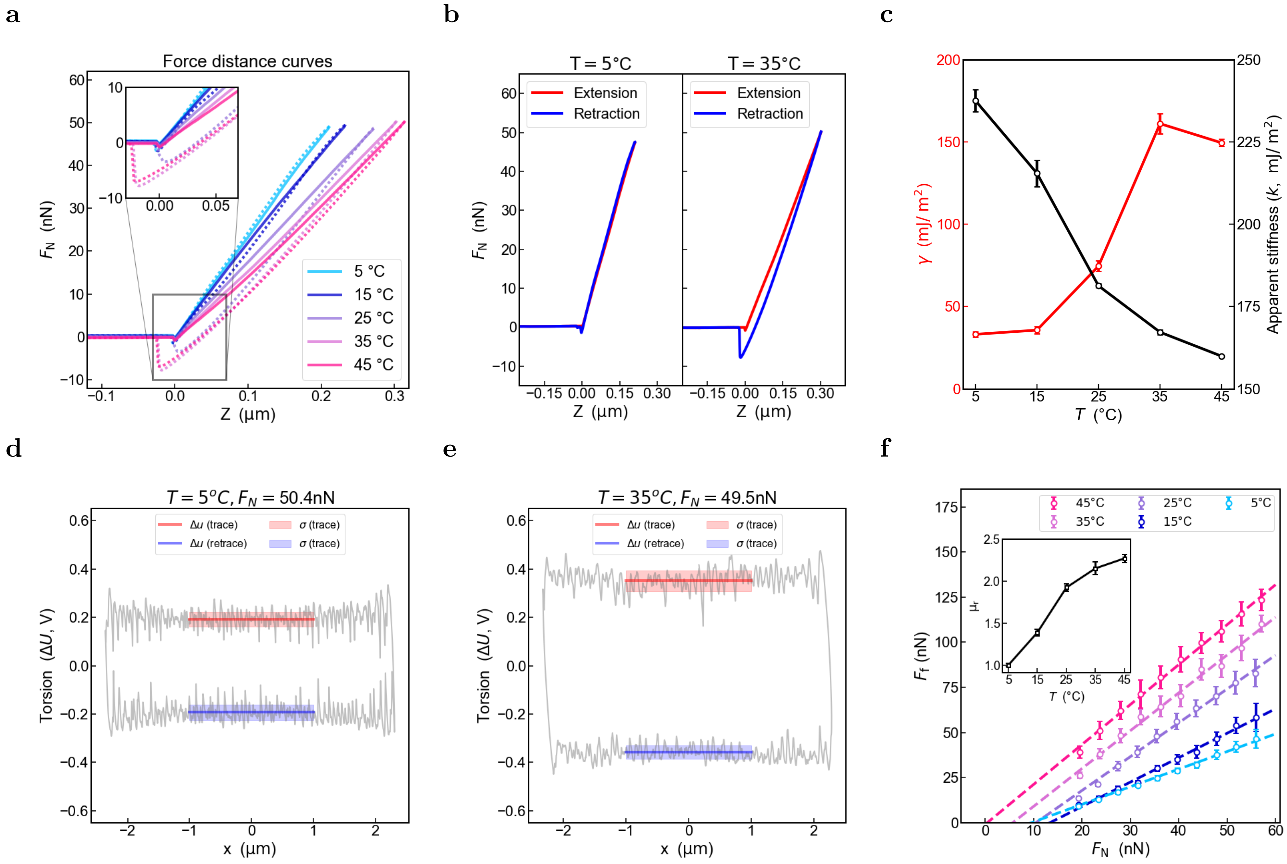

Supplementary Fig. S 15: **Normal and friction force measurements of P-9 film via AFM.** (a) Force distance curves taken by the PPP-LFMR silicon probe on the **P-9** polymer surface are highly temperature sensitive. The solid lines show that force curves of the probe moving towards the surface (extension) and the dashed lines show the probe moving away from the curve (retraction). In (b), the force distance curves at 5 °C is compared to those taken at 35 °C. (c) The effective surface energy is calculated as $\gamma = F_{ad}/3\pi R$ where F_{ad} is the minimum of the retraction curves in (a). The apparent stiffness is extracted from the slope of the linear part in the extensions curves in (a). The error bars represent the standard deviation of more than 10 measurements. (d-e)Representative friction loops at 5 °C and 35 °C respectively. The friction is proportional to the difference in torsion signal I, where $I = \Delta u(trace) - \Delta u(retrace)$. The scanning distance was 5μm and the middle 2μm (as highlighted in red and blue) was used for this calculation to exclude effects of stiction as the tip turns. (f) Friction v.s. normal force for all 5 temperatures. Inset plot shows the relative friction coefficient normalized by the lowest value: μ_r is computed as $\mu_r = \mu(T)/\mu(5^o\text{C})$.

Discussion

It can be seen from Fig S15.a that the force-distance curves are highly temperature dependent. At temperatures above 15 °C, the extension (approaching) and retraction (withdrawing) curves deviate significantly, implying surface deformability and viscoelasticity. This difference is demonstrated in (b) where the force distance curves at 5 °C is compared to those taken at 35 °C. The apparent stiffness decreases with temperature, consistent with the DMA measurements (Fig S15.c). In the mean time, the adhesion force F_{ad} extracted from the minimum of the retraction curves in (a) increases around 15°C. This behavior is typical for polymer materials: as the material transitions through T_g, polymer chain

dynamics are "activated" and the polymer chain relaxation leads to larger contact area at the interface as well as stronger interactions.

Surface energy Due to the viscoelastic nature of polymer, γ generally depends on the rate of deformation $V \approx \dot{s}R$. The strain rate is denoted by $\dot{s}$ here to avoid confusion with surface energy. R is the radius of the contact area. Previous literature has revealed that the surface energy $\gamma(\dot{s}, T)$ obeys the time temperature superposition principle [10].
In general, the shape of the function has two possibilities: (1) If the deformation rate $De \sim \dot{s}\tau \approx 1$ is comparable to polymer relaxation rate, a bell-shape curve is observed with a peak near T_g. (2) If the deformation rate is much faster than the polymer relaxation rate ($De \gg 1$), the peak is pushed to higher temperature and the curve is stretched to an S-shaped appearance [2, 10].
The surface energy can be estimated from the adhesion forces (F_{ad}) in the AFM measurements. We follow the JKR formulation as suggested by Zeng & Israelachvili and the effective surface energy γ can be estimated as [10]

$$\gamma = \frac{F_{ad}}{3\pi R}$$

At the deformation rate of 2μm/s, the Deborah number is estimated to be high around bulk T_g ($De \approx 100$). For the dry film of P-9 (bulk T_g(DSC) = 9°C, T_g(DSC) = 12°C), it is observed that γ increases with T from around 30mJ/m^2 below T_g to 150 mJ/m^2 above T_g (Figure S15. c). The values agree with previous studies of polystyrene using SFA (where the surface curvature is well-defined) that reported receding surface energy to increase from 50 mJ/m^2 below T_g to 150 mJ/m^2 around T_g. The shape of the curve is S-shaped with a broad peak appearing around 35°C, which is higher than the bulk T_g. As mentioned above, the shift and the S-shape is consistent with literature data where the deformation rate is high[2, 10]. Since the Deborah number at the particle interface in the suspension is different from the AFM conditions measured here, it is anticipated that there will be deviations from the the surface energy plotted above.

Friction Friction was measured from the torsion signal by scanning the probe at 90° to the cantilever. It can be seen that with the same normal force, the friction differs at 5°C and 35°C (Fig S15.d and e). By varying the normal force, the relative friction coefficients can be extracted (Fig S15.f). In this case, it is observed that the friction coefficient increases with temperature and seems to plateau at higher temperature. This is consistent with literature studies on poly(styrene) surface[2]. However, in general, the friction force depends on the deformation rate and solvent environment. Our experiments that utilize a dry film to provide an demonstration of the general trend. It takes more advanced techniques to fully reproduce the real inter-particle interactions in a suspension environment and get a quantitative understanding of the competition between deformability and adhesion relevant for the strong shear thickening rheology.
Friction and adhesion forces around T_g is a well-studied subject and various techniques have been employed in the literature. Dedicated and exquisite measurements have been performed using surface force apparatus (SFA)and scanning force microscopy (SFM). Readers interested in this subject can find systematic studies from Israelachvili and Overney [2, 10, 11].

Deborah number

The Deborah number defined as $De = \tau_{polymer}/\tau_{shear}$ is a dimensionless number that compares the polymer relaxation time scale to the deformation time scale. Normally, the characteristic time $\tau_{polymer}$ is the terminal relaxation time or Rouse time in linear polymers. Unlike linear polymers, there are some ambiguities in the definition of a characteristic time of a crosslinked polymer. We assume that the dangling chain length at the interface is close to the chain length between crosslinks.[12] From $E'(T)$ in Figure 2 which was measured at an oscillation frequency of $f = 1$Hz, $\tau_{polymer}$ can be determined for the temperature at which E' plateaus.

Taking P-9 as an example, the elastic modulus E' reaches the rubbery plateaus at around 15°C in the wet state, the polymer relaxation timescale is calculated from the oscillation frequency.

$$\tau_{polymer}(15°\text{C}) \approx 1/2\pi f \approx 0.16\ s$$

For the shear time scale, we estimate $\tau_{shear} = 1/\dot{\gamma}$. Here we pick $\phi = 51.5vol\%$ so that similar volume fractions can be compared for the other two chemistries. The average shear rate at 15°C in the thickening regime is found to be 8 s^{-1} from Figure. S10 d. The results for P-29 and P-50 are shown in the Table S1 below. The Deborah numbers are close to 1 from these calculations supporting our statement that surface relaxation time scales are comparable to deformation time scales.

Table S1. Estimation of Deborah numbers

Name	ϕ	T [°C]	f [Hz]	$\tau_{polymer}$ [s]	$\dot{\gamma}[s^{-1}]$	τ_{shear} [s]	De
P-9	51.5	15.00	1.00	0.16	8.00	0.13	1.27
P-29	50.1	25.00	1.00	0.16	12.00	0.08	1.91
P-50	51.4	45.00	1.00	0.16	25.00	0.04	3.98

We think the Deborah number is directly connected to the "constraint" and three regimes can be identified.

1. At temperatures way below T_g, the polymer is glassy and $\tau_{polymer}$ is extremely high and $De \gg 1$. This means that the dynamics are effectively frozen at the time scale of the deformation. This corresponds to the rigid particle limit with low friction/adhesion forces but high stiffness.

2. Around T_g (plus some shift), $\tau_{polymer}$ decreases dramatically and we expect to see a cross-over regime $De \sim 1$. The dissipative interactions happen at time scales comparable to the suspension shear flow, and this can lead to enhanced adhesion/frictions and to the highest constraints on inter-particle motions.

3. At temperatures well above T_g the polymer chain mobility further increases and $De \ll 1$. This produces liquid-like dynamics at the interface during the deformation and it corresponds to very low constraint on the particle motions.

The analysis above is greatly simplified as the characteristic shear rate depends on the stress and also varies with the volume fraction. Therefore, one temperature does not necessarily correspond to one Deborah number.

Suspension pull test

a **b**

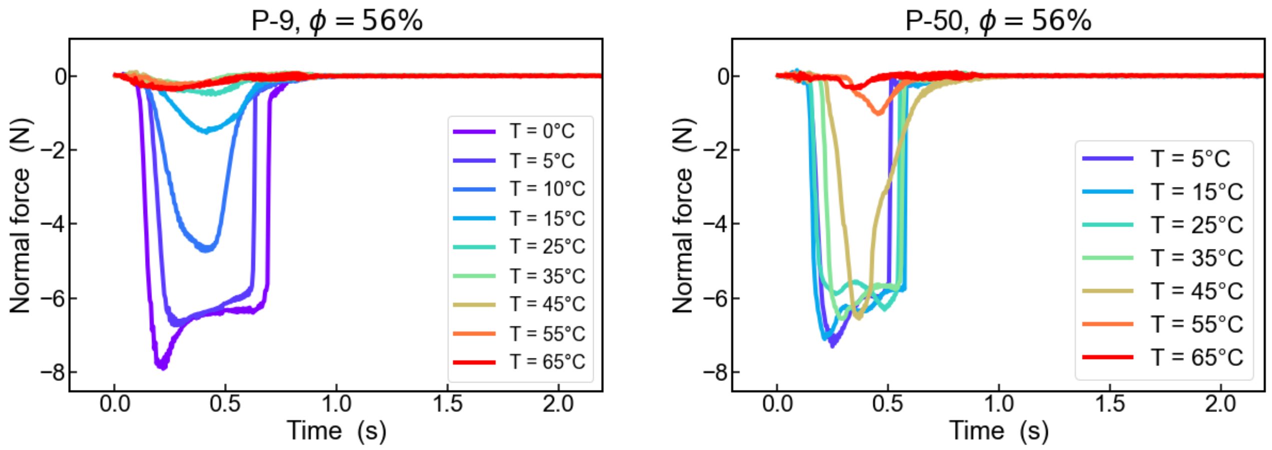

Supplementary Fig. S 16: **Representative normal force versus time curves via pull tests.** Representative raw data for the (a) P-9 and (b) P-50 suspensions ($\phi = 56\%$) shown in Fig. 4b and 4d, showing the normal force measured by the rheometer as the rod was pulled upwards at a rate of 8 mm s^{-1}. Significant dependence on temperature and differences between the **P-9** and **P-50** suspensions can be seen.

References

1. Buenviaje, C. K., Ge, S. R., Rafailovich, M. H. & Overney, R. M. Atomic force microscopy calibration methods for lateral force, elasticity, and viscosity. *Materials Research Society Symposium - Proceedings* **522,** 187–192 (1998).
2. Sills, S., Gray, T. & Overney, R. M. Molecular dissipation phenomena of nanoscopic friction in the heterogeneous relaxation regime of a glass former. *The Journal of Chemical Physics* **123,** 134902 (2005).
3. James, N. M., Han, E., de la Cruz, R. A. L., Jureller, J. & Jaeger, H. M. Interparticle hydrogen bonding can elicit shear jamming in dense suspensions. *Nature Materials* **17,** 965–970 (2018).
4. Lee, W. P. & Routh, A. F. Why do drying films crack? *Langmuir* **20,** 9885–9888 (2004).
5. Fischer-Cripps, A. C. *Introduction to Contact Mechanics* 101–115 (Springer US, Boston, MA, 2007).
6. Mari, R. & Seto, R. Force transmission and the order parameter of shear thickening. *Soft Matter* **15,** 6650–6659 (2019).
7. Rathee, V., Blair, D. L. & Urbach, J. S. Localized stress fluctuations drive shear thickening in dense suspensions. *Proceedings of the National Academy of Sciences* **114,** 8740–8745 (2017).
8. Singh, A., Ness, C., Seto, R., de Pablo, J. J. & Jaeger, H. M. Shear thickening and jamming of dense suspensions: the "roll" of friction. *Physical Review Letters* **124,** 2–6 (2020).
9. Živković, N., Šerbanović, S., Kijevčanin, M. & Živković, E. Volumetric properties, viscosities, and refractive indices of the binary systems 1-butanol, PEG 200, PEG 400, and TEGDME. *International Journal of Thermophysics* **34,** 1002–1020 (2013).
10. Zeng, H., Maeda, N., Chen, N., Tirrell, M. & Israelachvili, J. Adhesion and Friction of Polystyrene Surfaces around T g. *Macromolecules* **39,** 2350–2363 (2006).
11. Knorr, D. B., Gray, T. O. & Overney, R. M. Cooperative and submolecular dissipation mechanisms of sliding friction in complex organic systems. *The Journal of Chemical Physics* **129,** 074504 (2008).
12. Liu, S. *et al.* Brittle-to-Ductile Transition of Sulfonated Polystyrene Ionomers. *ACS Macro Letters* **10,** 503–509 (2021).
